# Iron–Inflammasome Crosstalk in Adipose Tissue: Unresolved Roles of NLRP3 and IL-1β in Metabolic Inflammation

**DOI:** 10.3390/ijms26178304

**Published:** 2025-08-27

**Authors:** Sixtus Aguree

**Affiliations:** Department of Applied Health Science, Indiana University School of Public Health, Bloomington, IN 47405, USA; saguree@iu.edu

**Keywords:** iron metabolism, NLRP3 inflammasome, IL-1β, adipose tissue, obesity, type 2 diabetes, ferroptosis, hepcidin, ferritin, metabolic inflammation

## Abstract

Iron is essential for cellular respiration, oxidative defense, and host immunity, but its dysregulation is increasingly associated with metabolic disorders, such as obesity and type 2 diabetes. In these diseases, regional iron accumulation occurs in adipose tissue, independent of systemic overload. This process disrupts the mitochondrial redox balance, induces ferroptotic stress, and activates the innate immune pathways. Recent studies have highlighted the NLRP3 (nucleotide-binding domain, leucine-rich repeat, pyrin domain-containing protein 3) inflammasome and its effector cytokine interleukin-1β (IL-1β) as important mediators of the interface between iron and inflammation. In both adipocytes and macrophages, labile iron increased reactive oxygen species (ROS) production and promoted inflammasome formation. Simultaneously, metabolic stress factors upregulate hepcidin expression, suppress ferroportin activity and exacerbate intracellular iron retention. These molecular events converge to maintain low-grade inflammation and impair insulin signaling. Despite these compelling associations, direct mechanistic evidence remains limited, particularly with respect to depot-specific responses and cell type resolution. In this review, I examine the current evidence linking iron handling and inflammasome biology in adipose tissue, focusing on ferroptosis, thioredoxin-interacting protein (TXNIP) signaling, and spatial mapping of iron–cytokine networks. I also discuss novel therapeutic strategies targeting iron overload and inflammasome activation, including chelation, hepcidin modulation, and inflammasome inhibition in the context of metabolic diseases.

## 1. Introduction

Obesity and type 2 diabetes mellitus (T2D) continue to be pressing global health problems, affecting over 890 million and 500 million people, respectively [1,2]. Although both conditions are traditionally characterized by excessive caloric intake and energy imbalance, they are increasingly recognized as systemic inflammatory conditions characterized by persistent, low-grade immune activation, commonly referred to as “metaflammation” [3,4,5,6]. Immunological remodeling is particularly evident in adipose tissue, where the proliferation of hypertrophic fat cells, local hypoxia, and monocyte recruitment remodels the tissue into a pro-inflammatory niche, promoting insulin resistance and impaired metabolic flexibility [7,8,9,10,11,12,13,14,15]. At the center of this immunometabolic shift is the NLRP3 (nucleotide-binding domain, leucine-rich repeat, pyrin domain-containing protein 3, often shorten to NOD-, LRR-, and pyrin domain-containing protein 3) inflammasome, a cytosolic sensing complex activated by metabolic danger signals, such as saturated fatty acids, reactive oxygen species (ROS), and other damage-associated molecular patterns (DAMPs). After priming and activation, NLRP3 facilitates autocatalytic cleavage of pro–caspase-1, thereby converting pro–interleukin-1β (pro–IL-1β) to its mature, biologically active form [6,16,17,18]. This metabolic pathway has been directly linked to local and systemic metabolic inflammation in obesity and insulin resistance [16,19,20,21,22,23,24].

At the same time, disturbances in iron homeostasis have been shown to be important, but underestimated, factors in adipose tissue inflammation. Iron is essential for the oxygen supply, mitochondrial electron transport, and enzymatic redox processes [25,26]. However, excess iron, particularly in the ferrous form (Fe^2+^), can promote the formation of hydroxyl radicals (OH) via Fenton chemistry, contributing to widespread oxidative damage to DNA, lipids, and proteins [27,28]. Oxidative damage is not harmless; it triggers ferroptosis, an iron-dependent form of regulated cell death that is increasingly associated with adipocyte attrition and metabolic disorders [29,30,31]. In fat depots, iron overload impairs mitochondrial respiration, suppresses key insulin-sensitizing adipokines, such as adiponectin and leptin, and reprograms resident macrophages to an inflammatory phenotype [32,33,34,35].

Furthermore, mitochondrial dysfunction in conjunction with excess iron can drive NLRP3 activation through ROS production and release oxidized mitochondrial DNA, providing a molecular bridge between iron dysregulation and cytokine-mediated inflammation [36,37,38].

An intriguing clinical paradox occurs in many people with obesity and T2D, and elevated serum ferritin levels often occur concurrently with normal or low transferrin saturation, a pattern referred to as dysmetabolic iron overload syndrome (DIOS) or metabolic hyperferritinemia [39,40]. These iron biomarkers indicate intracellular iron deposition rather than classic systemic iron overload. Recent studies have shown a disproportionate accumulation of iron in visceral adipose tissue (VAT), particularly in iron-containing macrophages, which exhibit increased NLRP3 expression and contribute to impaired glucose homeostasis [41,42].

Despite growing interest, many mechanistic gaps remain. Few studies have clarified how iron handling, including ferritin storage, ferroportin export, and hepcidin regulation, modulate the priming or activation of inflammasomes in adipose tissue. Furthermore, the roles of ferroptosis and iron-mediated oxidative stress in the maintenance of inflammatory signals have not been sufficiently characterized [34,43]. This review summarizes the current mechanistic and translational literature on iron–inflammasome interactions in adipose tissue. I evaluated the roles of redox imbalance, ferroptosis, iron trafficking, and macrophage–adipocyte crosstalk in the modulation of inflammation and insulin resistance. Finally, I explored therapeutic options targeting iron metabolism or inflammasome signaling, such as iron chelation, hepcidin antagonism, and interleukin-1 beta (IL-1β) blockade, as potential interventions to treat adipose tissue dysfunction in obesity and T2D.

## 2. NLRP3 Inflammasome: Activation and Function

NLRP3 inflammasome is a cytosolic multiprotein complex that plays a critical role in innate immunity and regulation of inflammation, particularly in the context of metabolic diseases such as obesity and type 2 diabetes (T2D) [44,45,46]. This complex consists of the sensor molecule NLRP3, adaptor protein ASC (apoptosis-associated speck-like protein containing a CARD), and effector enzyme pro-caspase-1, which recognizes various endogenous danger signals and pathogen-associated molecular patterns (PAMPs) and triggers inflammatory responses [47,48,49]. The NLRP3 inflammasome functions as a cytoplasmic surveillance system capable of recognizing a variety of metabolic stressors. Its activation occurs through a well-characterized two-step process involving distinct priming and activation signals, an arrangement that provides regulatory checkpoints critical for preventing abnormal cytokine release [50,51,52]. The initial “priming” signal typically involves Toll-like receptor (TLR) ligands such as lipopolysaccharide (LPS), or cytokines such as tumor necrosis factor alpha (TNF-α) and IL-1β, which activate the nuclear factor kappa-light-chain-enhancer of activated B cells (NF-κB) [53,54,55,56].This transcription program upregulates important inflammasome components such as NLRP3, pro–IL-1β, and pro–IL-18 [45,57]. The subsequent “activation signal” arises from intracellular perturbations such as adenosine triphosphate (ATP)-induced potassium efflux, lysosomal destabilization, and accumulation of mitochondria-derived reactive oxygen species (mtROS), all of which serve as DAMPs [53,58,59]. These stimuli (second signal) trigger oligomerization of NLRP3 and recruitment of the adaptor protein ASC, leading to autocatalytic activation of caspase-1 [49,60,61,62,63]. Activated caspase-1 then cleaves pro–IL-1β and pro–IL-18 into their mature, biologically active cytokines. It also cleaves Gasdermin D (GSDMD), releasing its N-terminal fragment, which oligomerizes and forms pores in the plasma membrane. These pores facilitate the release of IL-1β and IL-18, which are potent pro-inflammatory mediators [45,64], and induce pyroptotic cell death [65,66]. In particular, IL-1β plays a central role in chronic inflammation underlying insulin resistance and glycemic dysregulation in obesity and T2D [44,45]. After binding to its receptor (IL-1R), IL-1β amplifies inflammation by triggering the expression of additional cytokines, such as TNF-α, thus reinforcing a feed-forward inflammatory loop [44,45]. This mechanism enables an intricate interplay between immune cells, especially macrophages and metabolic cells such as adipocytes, and drives adipose tissue inflammation during metabolic stress [45]. In visceral adipose tissue, IL-1β also mediates a pro-inflammatory loop between macrophages and CD4+ T cells via IL-22, which worsens insulin resistance and glycemic control [67]. Thus, the NLRP3–IL-1β axis represents an important molecular link that translates metabolic excess into systemic inflammation and organ dysfunction in various tissues in metabolic diseases.

A wide range of stimuli can serve as secondary signals to activate NLRP3 inflammasome. Among the best characterized is ion flux, including potassium (K^+^) efflux, commonly triggered by extracellular ATP via P2 × 7 receptors activation. Other contributing signals include calcium (Ca^2+^) mobilization from the endoplasmic reticulum, chloride (Cl^−^) efflux, and sodium (Na^+^) influx [68,69]. A molecular link is the redox-sensitive protein thioredoxin-interacting protein (TXNIP), which dissociate from thioredoxin during oxidative stress directly binds to NLRP3. Notably, TXNIP expression is elevated in response to high glucose conditions, connecting metabolic stress to inflammasome activation [57,70,71]. Metabolic lipids such as palmitate and ceramides promote inflammasome activation through mitochondrial stress and AMPK inhibition, while uric acid and cholesterol crystals, as well as aggregated IAPP in pancreatic islets, also serve as well-characterized activators [51,61,72,73,74]. Other modulators, such as the mitochondrial antiviral signaling protein (MAVS), microtubule dynamics, and the kinase NEK7, have also been implicated in facilitating inflammasome assembly of the inflammasome [74,75].

**Figure 1:**
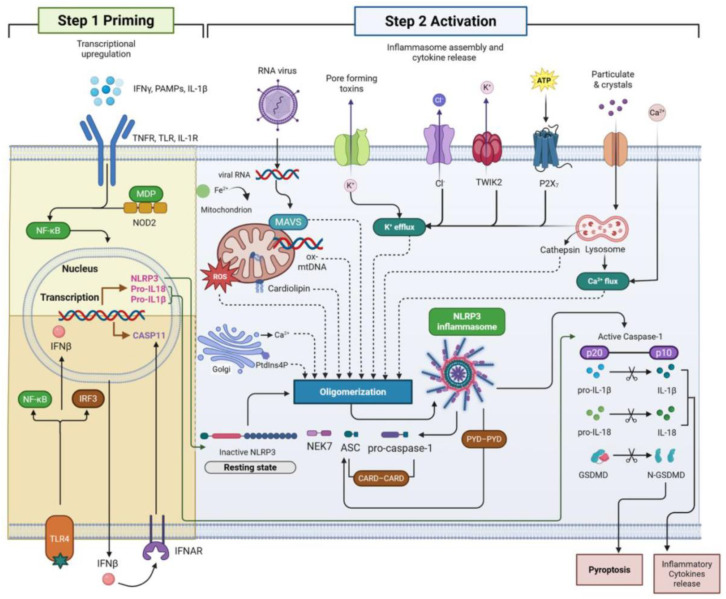
Molecular mechanisms of NLRP3 inflammasome priming and activation.

Overall, activation of the NLRP3 inflammasome is not a singular or isolated event but a finely tuned multi-signaling process that is closely intertwined with metabolic stress, immune cell activation, and inflammation (Figure 1). In the context of obesity and T2D, this process unfolds primarily in adipose tissue and pancreatic islets but also extends to vascular, hepatic, renal, and neural tissues, exacerbating systemic dysfunction. In the adipose tissue microenvironment, NLRP3 expression is normally minimal but is significantly increased under obese conditions, where chronic nutrient excess and insulin resistance are prevalent [22,23]. Obesity-associated stimuli such as lipotoxicity, mitochondrial dysfunction and increased extracellular ATP lead to activation of the NLRP3 inflammasome in both adipocytes and resident immune cells [29,31]. Recent evidence suggests that ferritinophagy—the autophagic degradation of ferritin—is a mechanistic link between iron metabolism and inflammasome activation. This process releases labile iron, enhances lipid peroxidation, and facilitates ferroptotic death, which further enhances NLRP3 activity [36,76]. These findings suggest that redox-active iron is an important upstream regulator of inflammasome signaling in metabolically stressed adipose tissue.

This figure illustrates the two-step model of NLRP3 inflammasome activation. Step 1 (priming) involves the transcriptional upregulation of key components of the inflammasome, such as NLRP3, pro-IL-1β, and pro-IL-18, mainly through NF-κB signaling activated by pattern recognition receptors (PRRs) such as TLRs (Toll-like receptors), NOD2, and cytokine receptors (e.g., IL-1R, TNFR). This step also involves the induction of IFN-β via IRF3, which further enhances immune responses. Step 2 (activation) is triggered by various cellular stress signals, including ATP, K^+^ efflux, ROS, mitochondrial dysfunction, lysosomal rupture, and ion fluxes (Cl^−^, Ca^2+^). (Iron overload and mitochondrial Fe^2+^ dysregulation enhance ROS production and release of oxidized mitochondrial DNA (mtDNA)—both potent NLRP3 activators). These events promote the conformational change and oligomerization of the NLRP3 inflammasome complex with ASC (apoptosis-associated speck-like protein containing a CARD), NEK7, and pro-caspase-1, leading to autocatalytic activation of caspase-1, which converts pro-IL-1β and pro-IL-18 to their mature forms and cleaves Gasdermin D (GSDMD), triggering pyroptotic cell death and the release of inflammatory cytokines. Thus, iron homeostasis directly modulates inflammasome sensitivity and inflammatory outcomes. Abbreviations: ASC, apoptosis-associated speck-like protein containing a CARD; CARD, caspase activation and recruitment domain; CASP1, caspase-1; DAMPs, damage-associated molecular patterns; GSDMD, Gasdermin D; IFN-β, interferon beta; IL-1R, interleukin-1 receptor; IRF3, interferon regulatory factor 3; MAVS, mitochondrial antiviral signaling protein; mtDNA, mitochondrial DNA; NEK7, NIMA-related kinase 7; NF-κB, nuclear factor kappa-light-chain-enhancer of activated B cells; NLRP3, NOD-like receptor family pyrin domain-containing 3; P2 × 7, purinergic receptor P2 × 7; PAMPs, pathogen-associated molecular patterns; PYD, pyrin domain; ROS, reactive oxygen species; TLR, Toll-like receptor; TNFR, tumor necrosis factor receptor; TWIK2, tandem pore domain weak inwardly rectifying K^+^ channel 2.

The NLRP3–IL-1β axis converts intracellular stress signals into extracellular inflammatory responses that impair insulin signaling, promote apoptosis of β cells, and maintain metabolic imbalance. Thus, the inflammasome acts as both a sensor and amplifier of metabolic dysfunction. Its central role in orchestrating chronic inflammation makes it a promising target for therapeutic intervention in metabolic diseases, particularly through inhibitors that suppress inflammasome assembly or cytokine release as well as dietary interventions aimed at mitigating upstream triggers. To synthesize the diverse molecular pathways by which iron influences inflammatory and metabolic processes, Table 1 presents a comparative overview of key mechanisms, including ferroptosis, hepcidin signaling, and macrophage–adipocyte iron flux, along with their associated mediators, affected tissues, and pathophysiological consequences in obesity and T2D.

## 3. NLRP3 and IL-1β in Obesity and Type 2 Diabetes

### 3.1. Mechanistic Role of the NLRP3–IL-1β Axis in Metabolic Inflammation

Obesity and T2D are characterized by chronic low-grade inflammation involving both innate and adaptive immunity [91,92]. At the center of this inflammatory process is the NLRP3 inflammasome, an intracellular sensor activated by a wide range of metabolic danger signals that accumulate in obesity, including saturated fatty acids (e.g., palmitate), ceramides, uric acid, high glucose, and islet amyloid polypeptide (IAPP) [45,64,72,93]. These signals promote the assembly and activation of inflammasomes, leading to the activation of caspase-1 and release of the pro-inflammatory cytokines IL-1β and IL-18. In adipose tissue, obesity leads to adipocyte hypertrophy and recruitment of immune cells, particularly adipose tissue macrophages (ATMs), which are a major source of NLRP3 expression and inflammasome activity [37,45,92,94]. The bidirectional interaction between macrophages and adipocytes leads to a pro-inflammatory environment that interferes with insulin signaling [95,96]. IL-1β impairs IRS-1 phosphorylation and inhibits insulin-stimulated lipogenesis in adipocytes [96], whereas caspase-1 inhibition promotes adipogenesis and improves insulin sensitivity in vitro and in obese mice [22]. Elevated IL-1β and IL-18 levels are consistently observed in the adipose tissue and serum of individuals with obesity and insulin resistance [64,67,97], and higher NLRP3 expression in visceral adipose tissue correlates with the severity of insulin resistance [45,67]. Importantly, NLRP3 inhibition, both genetic and pharmacological, has been shown to attenuate adipocyte hypertrophy induced by a high-fat diet and improve insulin signaling in adipose tissue, the liver, and skeletal muscle, which is often associated with increased Akt/PKB phosphorylation and improved glucose uptake [17,47,96,98,99].

In the pancreas, NLRP3 activation contributes to β-cell dysfunction—a defining feature of T2D. IL-1β plays a central role in the progressive decline of pancreatic β-cell function in both type 1 and type 2 diabetes. Evidence from human and animal studies suggests that IL-1β directly impairs glucose-stimulated insulin secretion, in part by inducing nitric oxide synthase and disrupting mitochondrial metabolism, thereby reducing insulin production in β-cells [100,101]. In addition, IL-1β suppresses the proliferation of β-cells by downregulating cell cycle regulators such as cyclin D2 and increasing the expression of cell cycle inhibitors including p21 and p27 [101,102]. Prolonged exposure to IL-1β also triggers apoptosis via activation of the nuclear factor-κB (NF-κB) and end oplasmic reticulum stress pathways, which promote the expression of pro-apoptotic proteins such as Bim and Bax [103,104]. The combined effects impaired insulin secretion, decreased regenerative capacity of β-cells, increased apoptotic death, and accelerated β-cell mass decline and functional failure, contributing to the onset and progression of hyperglycemia in diabetes [44,105,106].

NLRP3 is activated in the pancreatic islets and by infiltrating macrophages in response to IAPP oligomers that aggregate in the islets of patients with T2D [44,70]. These IAPP structures trigger inflammasome activation in macrophages and dendritic cells, further increasing IL-1β production and β-cell death [70,72]. Chronic hyperglycemia enhances NLRP3 activation by increasing the expression of thioredoxin-interacting protein (TXNIP) and the production of ROS, creating a forward loop of glucotoxicity (including paracrine and autocrine damage to β-cells) and inflammation [16,38,57,70,71]. In mouse models, genetic ablation of NLRP3 or ASC adaptor protects against the loss of β-cells, increases islet area, and improves insulin levels, highlighting the pathogenic role of the inflammasome in islet degeneration [48,107,108].

### 3.2. Tissue-Specific Inflammatory Pathways and Iron-Mediated Activation

In mice fed a high-fat diet (HFD), the expression of NLRP3 and IL-1β is significantly increased in visceral white adipose tissue (vWAT), which precedes the development of hyperglycemia and hyperinsulinemia. Genetic deletion of *nlrp3* or caspase-1 (*casp*)1 leads to resistance to diet-induced insulin resistance despite equal energy intake and weight gain, underscoring the causal role of the inflammasome [48,109]. This pro-inflammatory signaling is anatomically specific [110]. Visceral fat depots, especially epididymal and perigonadal, exhibit stronger inflammasome activity than subcutaneous adipose tissue, which is due to differences in stromal vascular composition, immune infiltration, and adipocyte size [4,5]. Crown-like structures (CLS), characterized by clusters of differentiation 11c (CD11c^+^) macrophages surrounding necrotic fat cells, are enriched in vWAT and correspond with impaired insulin signaling via reduced insulin receptor substrate 1 (IRS-1) phosphorylation and protein kinase b (PKB) (AKT) activation [111,112]. IL-1β acts as a versatile mediator of metabolic dysfunction. Mechanistically, it impairs insulin signaling by upregulating the suppressor of cytokine signaling (SOCS) proteins and activating c-Jun N-terminal kinase/mitogen-activated protein kinase (JNK/MAPK) cascades, thereby inhibiting insulin receptor substrate (IRS) function [113,114]. In human studies, increased IL-1β expression in adipose tissue correlates with insulin resistance independent of obesity [9,35]. Beyond glucose metabolism, IL-1β disrupts adipokine profiles by downregulating adiponectin and stimulating leptin secretion, which exacerbates systemic inflammation and metabolic imbalance [32,40,115]. Although macrophages remain the major source of IL-1β in adipose tissue, functional inflammasome machinery is also expressed in preadipocytes, mature adipocytes, dendritic cells, and mast cells, all of which contribute to a self-sustaining inflammatory cycle [94,109,116].

Iron metabolism overlaps with inflammasome signaling in several important nodes. Enlargement of the labile iron pool (LIP), often triggered by hepcidin-mediated ferroportin degradation, promotes mitochondrial ROS production and activates the NLRP3 inflammasome [29,41,42,117,118]. Iron chelation with deferoxamine and the human myelomonocytic cell line THP-1 has been shown to suppress the release of IL-1β and restore insulin sensitivity in obese mice and adipocyte cultures [36,119]. Inflammatory syndromes associated with iron dysregulation are increasingly recognized in clinical contexts of chronic iron overload. A prominent example is β-thalassemia, in which both transfusional iron loading and ineffective erythropoiesis contribute to the excessive release of intracellular iron into the plasma [120,121]. This leads to increased concentrations of non-transferrin-bound iron (NTBI), particularly in the Fe^2+^ state, which can catalyze Fenton reactions and generate ROS [122]. ROS initiate and propagate oxidative damage, leading to the activation of pro-inflammatory signaling pathways such as NF-κB [123,124]. This mechanism underlies the chronic inflammation observed in β-thalassemia and other iron-containing anemias and establishes a clear pathophysiological link between excess iron at the cellular iron and systemic inflammatory responses [121,125]. These effects highlight the potential of targeting excess iron as a therapeutic strategy for metabolic inflammation.

### 3.3. Therapeutic Approaches to Modulating NLRP3 Activity

Chelation therapy is a rational intervention to reduce iron-induced redox stress and interrupt inflammasome activation. Of the available agents, deferoxamine (DFO), deferiprone (DFP), and deferasirox (DFX) have shown efficacy in lowering labile iron and suppressing ROS production in preclinical models [122,126]. DFO, a hexadentate iron chelator, preferentially binds Fe^3+^ and reduces the availability of iron for Fenton chemistry, whereas DFP and DFX show better tissue penetration and prolonged plasma activity. These agents not only prevent iron accumulation in key metabolic organs such as the liver and heart but also blunt downstream inflammasome responses. In experimental models of dietary obesity, chelation with DFP and DFO has been shown to reduce IL-1β expression, normalize insulin signaling, and reduce macrophage infiltration into adipose tissue [36,127,128]. Additionally, chelators can indirectly stabilize mitochondrial function by preventing iron overload-induced depolarization and oxidative stress. The specificity of these agents for Fe^3+^ suggests that their anti-inflammatory effects are mediated by limiting labile iron availability and curbing Fe^2+^-driven formation of hydroxyl radical formation. Beyond inflammation, iron chelators improve metabolic parameters by restoring adipokine profiles and reversing insulin resistance [129,130]. These pleiotropic benefits make iron chelation a potential adjunct therapy for metabolic syndrome, particularly in individuals with subclinical iron overload or inflammatory hyperferritinemia. In combination with lifestyle and pharmacological interventions, iron detoxification may offer a multi-pronged approach to break the metabolic–inflammatory cycle driven by the NLRP3–IL-1β axis.

Taken together, these data demonstrate that the NLRP3–IL-1β axis is an important link between metabolic stress, redox imbalance, and immunological remodeling in adipose tissue. Its activation is modulated by depot location, iron availability, and mitochondrial integrity. Determining the temporal and spatial dynamics of this pathway may provide new therapeutic targets to interrupt the iron–inflammatory cycle in metabolic diseases. Promising therapeutic strategies targeting NLRP3 include small molecule inhibitors such as MCC950, which reduce systemic inflammation and improve glycemic control, and dietary interventions, such as n-3 polyunsaturated fatty acids (PUFAs) like DHA, which block saturated fat-triggered NLRP3 activation and insulin resistance [63,69,71,131,132,133]. Nevertheless, further research is needed to elucidate tissue- and cell-type-specific mechanisms, differences between canonical and non-canonical activation pathways, and relative contributions of central versus peripheral NLRP3 activity to systemic metabolic diseases.

## 4. Iron-Induced Inflammasome Activation: Molecular Pathways

The intersection of iron metabolism and inflammasome signaling represents a critical axis in the development of metabolic inflammation, particularly in adipose tissue under conditions of obesity and T2D. Chronic low-grade inflammation, also known as metaflammation, has emerged as a hallmark of insulin resistance and a defining feature of T2D pathogenesis [3]. At the center of this inflammatory cascade, the NLRP3 inflammasome is a cytosolic pattern recognition complex that integrates metabolic, redox, and inflammatory factors to drive *casp1* activation and IL-1β maturation [22,23]. Iron overload is increasingly being recognized as an upstream trigger of NLRP3 activation through its role in redox imbalance, mitochondrial dysfunction, and ferroptosis [134,135]. These iron-dependent processes promote the release of DAMPs, mitochondrial ROS, and oxidized lipids that activate inflammasomes. In particular, adipose tissue exhibits depot-specific susceptibility, with visceral adipose tissue (VAT) showing greater susceptibility to iron accumulation, oxidative damage, and inflammasome induction than subcutaneous and brown adipose tissues. The following subsections describe the major mechanisms by which iron disrupts adipose tissue (BAT) immune homeostasis, including ferroptosis, ferritinophagy, and thioredoxin-interacting protein (TXNIP).

### 4.1. Iron Overload and ROS

The ability of iron to switch between ferrous (Fe^2+^) and ferric (Fe^3+^) states makes it a strong catalyst for ROS via the Fenton reaction, which converts H_2_O_2_ into ·OH that can damage lipids, proteins, and DNA [136,137,138,139,140]. In lipid-rich environments, such as adipose tissue, this oxidative potential is amplified, particularly under conditions of diet-induced obesity where mitochondrial iron accumulation occurs. In the VAT, mitochondrial iron overload increases mtROS production, which serves as the initial signal for NLRP3 activation and subsequent IL-1β secretion [48,53,141]. Experimental models fed a high-fat diet (HFD) consistently showed increased expression of NLRP3 and downstream effectors in parallel with macrophage infiltration and systemic insulin resistance, linking mitochondrial iron dysregulation to metabolic disturbances [23]. These redox disturbances not only activate the inflammasome but also initiate a self-reinforcing loop of adipocyte dysfunction and immune cell recruitment. The increased sensitivity of NLRP3 to mtROS makes this axis particularly susceptible to dietary, genetic, and pharmacological modulation and provides a therapeutic target for intervention [56,142,143].

### 4.2. Ferroptosis and Ferritinophagy

Ferroptosis, a form of regulated necrosis characterized by iron-catalyzed lipid peroxidation, is increasingly being recognized as a central mediator of adipose tissue inflammation. Disruption of glutathione peroxidase 4 (GPX4) activity leads to the accumulation of lipid peroxide and the formation of cytotoxic aldehydes such as 4-hydroxynonenal (4-HNE), which compromise membrane integrity and release DAMPs [31,144,145]. DAMPs released during ferroptotic cell death serve as crucial mediators linking intracellular iron toxicity to immune activation. As lipid peroxidation progresses and membranes rupture, DAMPs such as high mobility group box 1 (HMGB1), ATP, mitochondrial DNA, and oxidized lipids are released into the extracellular environment. These signals encounter pattern recognition receptors such as TLR4, RAGE, and NLRP3 on immune cells, thereby enhancing NF-κB signaling, inflammasome formation, and the release of pro-inflammatory cytokines [146,147,148]. Among these, HMGB1 plays a particularly well-characterized role in ferroptosis, where its redox-dependent release enhances innate immune responses and promotes macrophage recruitment. This DAMP-driven inflammatory feedback loop amplifies local cytokine production, hepcidin expression, and further labile iron retention—ultimately reinforcing the iron–ROS–inflammatory axis. In contrast to ferritin-bound Fe^3+^, which is relatively inert, unstratified or labile Fe^2+^ drives continuous ROS formation, lipid peroxidation and DAMP release. This underlies the immunometabolic dysfunction observed in metabolic disease models with iron overload [129,130].

In adipocytes with ferroportin deficiency or excess iron, ferroptosis is accompanied by increased IL-1β secretion and NLRP3 activation, suggesting that ferroptotic cell death directly enhances inflammasome signaling. Pharmacological inhibitors of ferroptosis, including ferrostatin-1 and liproxstatin-1, have been shown to reduce IL-1β levels and improve insulin sensitivity in preclinical models [145,149]. This bidirectional association suggests that iron overload triggers ferroptosis and that the products of cell death serve as ligands for inflammasome activation. Furthermore, the regulation of ferroptosis overlaps with nutrient- and redox-sensing pathways, making it a modifiable node within a broader immunometabolic network. These findings suggest that ferroptosis is a therapeutically viable process linking iron dysregulation to chronic obesity. Thus, ferroptosis is a downstream manifestation of unbuffered Fe^2+^ reactivity that promotes sustained ROS production and inflammatory death [150,151].

A related amplification loop is ferritinophagy, the selective degradation of ferritin via the autophagy nuclear receptor coactivator 4 (NCOA4), which acts as a cargo receptor that binds directly to H-ferritin and facilitates its transport to lysosomes for degradation [152,153]. This interaction enables the controlled degradation of ferritin and subsequent release of stored iron into the cytosol [154,155]. The process is crucial for the mobilization of iron from intracellular stores and the release of iron into the cytosolic labile iron pool (LIP), making iron available for metabolic needs, especially in times of iron scarcity or increased demand [155,156,157,158,159]. This interaction ensures that ferritin is delivered to the lysosomes, where it is degraded, and the stored iron is released into the cytosol [155,160]. The C-terminus of NCOA4 contains a ferritin-binding domain, and this binding is specifically upregulated under conditions of iron deficiency, which are often promoted by iron chelators. NCOA4 also functions as an iron sensor [155]. Under high intracellular iron conditions, NCOA4 is degraded via polyubiquitination mediated by the E3 ubiquitin ligase HECT and RLD domain-containing E3 ubiquitin protein ligase 2 (HERC2) [154,161,162]. Conversely, NCOA4 accumulates during iron deficiency, binds to ferritin, and facilitates iron recycling [155]. This regulatory mechanism provides an additional level of control over cytosolic iron homeostasis and complements the well-established iron-dependent binding of Iron Regulatory Proteins (IRPs) to Iron Responsive Elements (IREs) [155].

Under physiological conditions, ferritinophagy supports iron recycling and mitochondrial functions. However, the upregulation of this pathway associated with obesity increases cytosolic Fe^2+^, which increases susceptibility to ROS formation and ferroptotic stress [36,163]. The resulting increase in labile iron levels promotes mitochondrial dysfunction, oxidative DNA damage, and the release of ATP and oxidized lipids, which are known triggers of NLRP3 activation. These processes are modulated by metabolic regulators such as AMP-activated protein kinase (AMPK), nuclear factor erythroid 2–related factor 2 (NRF2), and mammalian (or mechanistic) target of rapamycin (mTOR), which influence autophagic flux and redox balance [163]. Dysregulation of ferritinophagy has been associated with various metabolic complications including insulin resistance and hepatic steatosis [163]. Studies in preclinical models have shown that inhibiting ferritinophagy or blocking ferroptosis attenuates inflammasome activity and restores insulin sensitivity [79,145]. Therefore, ferritinophagy represents a key mechanistic pathway by which excess nutrients promote iron mobilization and immune activation in adipose tissue. Notably, ferritinophagy and ferroptosis may also reinforce each other through a deleterious positive feedback loop: the degradation of ferritin via NCOA4 releases labile Fe^2+^, which amplifies ROS production and lipid peroxidation, driving ferroptosis. This ferroptotic damage may, in turn, signal further ferritin degradation, perpetuating a self-reinforcing “vicious cycle” [149]. The clinical implications of this relationship are far from clear. Although the clinical implications of this relationship remain to be fully elucidated, targeting the NCOA4–ferritin axis offers a promising strategy to limit iron release and potentially prevent ferroptosis in metabolic and inflammatory diseases [158].

### 4.3. TXNIP and Mitochondrial Stress

Finally, the redox-sensitive adaptor thioredoxin-interacting protein (TXNIP) serves as an important integrator of oxidative stress and NLRP3 inflammasome activation. Under basal conditions, TXNIP binds to thioredoxin (TRX), an antioxidant enzyme that neutralizes ROS and maintains cellular redox homeostasis. During oxidative insult, particularly mitochondrial iron overload, TXNIP dissociates from TRX and binds directly to NLRP3, facilitating the assembly and activation of caspase-1 [56,142,143]. In adipose tissue, TXNIP expression is upregulated in response to a HFD and correlates with mitochondrial fragmentation, membrane depolarization, and increased IL-1β secretion [4]. Accumulation of oxidized mitochondrial DNA exacerbates this response by acting as a secondary danger signal for the inflammasome complex. Genetic ablation of TXNIP in mouse models results in decreased NLRP3 activation, improved glucose homeostasis, and attenuated adipose tissue inflammation, confirming its role as a hub in the iron–ROS–inflammasome axis [163]. Together, these interconnected pathways, including ROS formation, ferroptotic cell death, ferritinophagy-driven iron flux, and TXNIP signaling, form a tightly coupled network of iron-induced immunometabolic stress. To support the mechanistic framework described above, various experimental models, ranging from transgenic mice to co-culture systems and human adipose biopsies, have been employed to dissect the iron–inflammasome axis in metabolic disease contexts. Table 2 summarizes key in vivo, in vitro, and clinical studies that have established the biological relevance and translational potential of iron-mediated inflammasome activation in obesity and type 2 diabetes.

## 5. Systemic and Local Iron Metabolism

### 5.1. Physiological Iron Handling and Cellular Regulation

**Figure 2:**
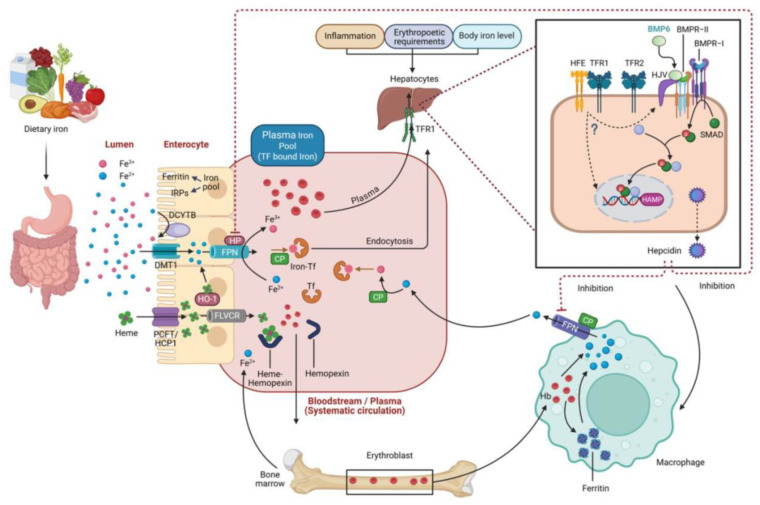
Integrated regulation of dietary iron absorption and systemic iron homeostasis.

Iron is essential for numerous biological processes including oxygen transport, oxidative phosphorylation in the mitochondria, DNA synthesis, and host immune defense [25,26]. Dietary iron absorption predominantly occurs in the duodenum, where iron from food, mainly in the Fe^2+^ form, is transported into enterocytes via the divalent metal transporter 1 (DMT1). Non-heme iron ingested with food is usually absorbed as Fe^3+^, which must first be reduced to Fe^2+^ by duodenal cytochrome b (Dcytb) prior to transport. Once inside the enterocyte, Fe^2+^ may be stored intracellularly in ferritin or exported across the basolateral membrane via ferroportin. During export, Fe^2+^ is re-oxidized by ferroxidases such as hephaestin and ceruloplasmin to enable its binding to transferrin (Tf) in the plasma, ensuring that redox-active Fe^2+^ remains transient and tightly regulated [158]. Cellular uptake of transferrin-bound iron occurs via transferrin receptor 1 (TfR1)-mediated endocytosis. Within acidified endosomes, Fe^3+^ is released from transferrin, reduced to Fe^2+^, and transported into the cytosol by DMT1. Cytosolic iron is directed toward mitochondrial heme synthesis and the formation of iron–sulfur (Fe–S) clusters or stored in ferritin (Figure 2). A portion remains in the labile iron pool (LIP), a metabolically active but redox-reactive fraction that must be carefully regulated [169]. Ferritin, a 24-subunit heteropolymer, is capable of safely storing up to 4500 Fe^3+^ atoms in a mineral core [156,170], serving as both a buffer reservoir and a protective mechanism against oxidative stress. Macrophages, particularly those within the reticuloendothelial system, recycle iron from senescent erythrocytes through erythrophagocytosis and subsequent heme degradation, a process mediated by heme oxygenase-1 (HO-1), which releases bioavailable iron while limiting pro-oxidant heme accumulation [43,171].

This figure provides a comprehensive overview of the absorption, distribution, and systemic regulation of iron. Dietary iron is absorbed in the duodenum as non-heme (Fe^3+^) and heme iron. Non-heme Fe^3+^ is reduced to Fe^2+^ by DCYTB and transported into enterocytes via DMT1. Heme uptake is more complex. HCP1, now recognized as the PCFT, was once proposed as the primary heme transporter; however, PCFT has higher affinity for folate, making its physiological role in heme absorption limited. Intracellular heme is degraded by heme oxygenases (HO-1/HO-2) to release Fe^2+^, with HRG1 mediating lysosomal heme transport and FLVCR1 exporting excess heme to prevent toxicity. Within enterocytes, Fe^2+^ is stored as ferritin or exported via ferroportin (FPN). Fe^2+^ is exported through the basolateral membrane by ferroportin (FPN) and oxidized primarily by hephaestin (HP) and secondarily by ceruloplasmin (CP), allowing Fe^3+^ to be loaded onto transferrin (Tf) in plasma for systemic distribution. Tf-bound Fe^3+^ is taken up by the target cells via transferrin receptors (TfR1, TfR2); endosomal STEAP3 reduces Fe^3+^ to Fe^2+^, which exits through DMT1. Systemic regulation is primarily controlled by hepcidin, which is synthesized in hepatocytes and regulated by BMP/SMAD signaling involving BMP6, HJV, HFE, and TFR2. Hepcidin inhibits iron export by inducing the degradation of FPN, thereby lowering plasma iron levels. Macrophages recycle iron from senescent red blood cells, by storing it as ferritin or exporting it via FPN, contributing to the circulating iron pool. Cellular iron metabolism is further regulated by iron regulatory proteins (IRPs), which control the expression of DMT1, FPN, ferritin, and TfR based on intracellular iron status. Additionally, FLVCR exports excess intracellular heme, particularly in erythroid cells, to prevent heme-induced toxicity. Together, these mechanisms coordinate dietary iron absorption, systemic distribution, erythropoietic demand, inflammation, and storage to maintain iron balance and prevent deficiency or overload. Abbreviations: BMP6, bone morphogenetic protein 6; CP, ceruloplasmin; DCYTB, duodenal cytochrome B; DMT1, divalent metal transporter 1; Fe^2+^/Fe^3+^, ferrous/ferric iron; FLVCR, feline leukemia virus subgroup C receptor; FPN, ferroportin; HAMP, hepcidin antimicrobial peptide; Hb, hemoglobin; HCP1, heme carrier protein 1; HFE, hemochromatosis protein; HJV, hemojuvelin; HO-1, heme oxygenase-1; HP, hephaestin; IRPs, iron regulatory proteins; PCFT, proton-coupled folate transporter; SMAD, mothers against decapentaplegic homolog; Tf, transferrin; TfR1/2, transferrin receptor 1/2.

Iron homeostasis in mammalian cells is predominantly controlled by the iron regulatory protein (IRP)–iron responsive element (IRE) post-transcriptional system, which modulates the translation and stability of mRNAs encoding key proteins involved in iron uptake, storage, and export in response to fluctuations in intracellular iron levels [43,162,172,173,174,175]. Under low intracellular iron conditions, IRP1 and IRP2 bind to IRE stem-loop motifs located in the untranslated regions (UTRs) of the target transcripts. Interaction with 5′-UTR IREs inhibits the initiation of translation of mRNAs encoding ferritin, ferroportin, ALAS2, and HIF-2α, thereby reducing iron storage and export. Conversely, binding to 3′-UTR IREs stabilizes transcripts such as transferrin receptor 1 (TfR1) and divalent metal transporter 1 (DMT1), prolonging their half-life and facilitating increased iron uptake [176,177,178]. Together, these actions enhance iron uptake and limit iron storage and export. When intracellular is sufficient, IRP1 assembles a [4Fe–4S] cluster and adopts a cytosolic aconitase function, resulting in a loss of RNA-binding activity [179,180], promoting ferritin synthesis and ferroportin-mediated export while descreasing TfR1/DMT1 expression. In contrast, IRP2, which lacks an Fe–S cluster, is degraded via FBXL5-mediated ubiquitination in response to elevated intracellular iron [181].

At the interface of cellular and systemic iron regulation, ferroportin is the sole identified iron exporter, which is subject to dual control: translational repression via IRPs and post-translational downregulation by the hepatic peptide hormone hepcidin [182]. Through its influence on intracellular iron pools and key iron-handling proteins, such as TfR1 and HIF-2α, the IRP–IRE system indirectly modulates hepcidin transcription. In duodenal enterocytes, HIF-2α serves as a transcriptional activator of genes encoding DMT1, duodenal cytochrome B, and ferroportin during dietary iron deficiency [183]. IRP1 attenuates this absorptive response by suppressing HIF-2α translation, thereby fine-tuning iron uptake [184,185]. Review of systemic and cellular homeostasis are published elsewhere [43,158,160,174,175,186,187].

### 5.2. The Hepcidin–Ferroportin Axis and Systemic Control

Unlike other micronutrients, iron does not have a regulatory excretion mechanism in humans. As a result, the iron balance throughout the body is maintained by a finely tuned system that includes intestinal absorption, tissue storage, and macrophage-mediated recycling of senescent erythrocytes [176]. Central to this regulatory network is the hepcidin–ferroportin axis. Hepcidin, a 25 amino acid hepatic peptide, acts as a major hormonal regulator of iron excretion by binding to ferroportin (FPN), the only known iron exporter in mammals, and is encoded by SLC40A1 [188,189]. Binding to hepcidin initiates the internalization and lysosomal degradation of FPN. The process limits the release of iron from duodenal enterocytes, hepatocytes, and macrophages into the plasma compartment [26,176,190,191]. Hepcidin regulation is multifactorial and responds to both systemic and local factors. Hepcidin expression is upregulated by the bone morphogenetic protein 6–SMAD family member 1/5/8 (BMP6–SMAD1/5/8) signaling cascade in response to systemic iron loading and is suppressed by an erythropoietic requirement via erythroferron secreted by erythroblasts in the bone marrow [192]. Hypoxic conditions, which often occur in metabolically stressed tissues, downregulate hepcidin via hypoxia-inducible factors (HIFs), thereby increasing iron availability. Inflammatory signals, particularly interleukin-6 (IL-6), increase hepcidin transcription by activating the Janus kinase–signal transducer and activator of the transcription 3 (JAK–STAT3) pathway [120,192,193].

### 5.3. Pathological Dysregulation: Overload, Inflammation, and Metabolic Impact

Perturbations in these regulatory networks can result in iron accumulation or hyperferremia. In primary or genetic iron overload, such as hereditary hemochromatosis, mutations in HFE, HJV, TFR2, or SLC40A1 impair hepcidin signaling, leading to unregulated intestinal iron absorption. As transferrin saturation exceeds 60%, NTBI, which is rich in Fe^2+^, appears in circulation and bypasses transferrin-mediated uptake, accumulating in the liver, pancreas, and heart muscle [160]. In secondary or acquired iron overload, repeated blood transfusions (as in thalassemia or myelodysplasia), high-dose parenteral iron administration, or hemolytic anemias introduce excess iron into the bloodstream. Once transferrin become saturated, labile Fe^2+^ accumulates in the tissues leading to oxidative damage [194]. Dietary and gut barrier factors can further modulate systemic iron metabolism. Diets high in carbohydrates and protein, as often observed in obesity and T2D, are associated with increased intestinal permeability (“leaky gut”), which facilitates the translocation of bacterial components such as lipopolysaccharide (LPS) into the bloodstream [195,196]. These microbial components activate pattern recognition receptors such as Toll-like receptor 4 (TLR4) in adipose tissue macrophages, stimulating cytokine-mediated hepcidin production and promoting intracellular iron sequestration [197,198,199]. The resulting increase in labile Fe^2+^, combined with inflammatory signaling, amplifies oxidative stress and links dietary patterns, gut permeability, and iron-driven inflammation in metabolic disease.

Pro-inflammatory cytokine signaling also contributes to hyperferritinemia independent of true iron overload. Cytokines such as IL-6 and IL-1β induce ferritin expression as part of the acute phase response, masking latent iron deficiency. Simultaneously, cytokine-mediated suppression of ferroportin, through both hepcidin-dependent degradation and transcriptional repression, limits iron efflux from enterocytes and macrophages, leading to hypoferremia and anemia of chronic disease [198,200]. During inflammation, iron metabolism is actively reprogrammed as part of a host defense strategy known as “nutritional immunity,” whereby iron is removed from the bloodstream to limit microbial growth [200,201]. IL-6 serves as a key inflammatory mediator that drives hepcidin induction via the STAT3 signaling cascade, leading to transcriptional upregulation of hepcidin antimicrobial peptide (HAMP) and increased iron storage [190]. IL-1β also exerts a potent effect, particularly in metabolic tissues, where it activates the NF-κB and p38 MAPK (mitogen-activated protein kinase) signaling pathways to induce hepcidin independent of IL-6 [202,203]. Additional cytokines, including TNF-α and interferons, suppress erythropoietin production and directly impair erythroid progenitor cell survival, further exacerbating erythropoietic dysfunction [39]. Together, these in turn increase ferritin synthesis, particularly in macrophages, independent of true iron sufficiency, thereby increasing serum ferritin concentration and masking latent iron deficiency [204]. At the same time, ferroportin expression is suppressed by hepcidin-dependent degradation and cytokine-mediated transcriptional repression, resulting in reduced iron efflux from enterocytes and macrophages [120]. While this iron-limiting phenotype is protective in acute infection, it becomes maladaptive during chronic inflammation. In conditions like obesity and T2D, persistent low-grade inflammation disrupts the hepcidin–ferroportin cycle, contributing to iron misdistribution, functional iron deficiency, and anemia of chronic disease (ACD)/anemia of inflammation (AI) [205]. Chronic inflammation also promotes iron sequestration into multiple tissues including the liver, spleen, bone marrow, pancreas, and heart through hepcidin-mediated ferroportin suppression and macrophage retention, leading to oxidative stress, tissues dysfunction, functional deficiency, and ACD/AI [39,206]. This results in a biochemical profile defined by low serum iron, decreased transferrin saturation, elevated ferritin, and impaired erythropoiesis [120].

Although these regulatory dynamics are well recognized in infections and autoimmune diseases, their importance in metabolic inflammation is increasingly appreciated. In obesity, persistent low-grade inflammation interferes with the hepcidin–ferroportin cycle, contributes to systemic iron maldistribution and impaired oxygen utilization in metabolically active tissues, and contributes to adipose tissue dysfunction and systemic metabolic dysfunction [33]. The immunometabolic effects of iron dysregulation vary considerably across cell types and adipose tissue depots. As summarized in Table 3, adipocytes, adipose tissue macrophages (ATMs), hepatocytes, and insulin-sensitive tissues exhibit distinct iron-handling characteristics that collectively shape tissue-specific outcomes of inflammation, ferroptosis, and insulin resistance in obesity and T2D.

## 6. Depot-Specific Iron Signaling in Adipose Tissue

Adipose tissue has been shown to be an iron-sensitive metabolic organ that exhibits different regulatory mechanisms and inflammatory reactions in different anatomical depots. Both adipocytes and adipose tissue macrophages (ATMs) express core components of the iron-processing machinery, including TfR1, DMT1, ferritin, FPN, and hepcidin. Under normal conditions, this network coordinates iron uptake, sequestration, and export to maintain redox homeostasis and cellular functions. However, in obesity and T2D, these regulatory hubs are disrupted, primarily due to chronic low-grade inflammation and IL-6–induced hepcidin upregulation, which promotes FPN degradation and the associated intracellular iron binding, as described above. This shift in iron dynamics not only alters systemic iron availability but also leads to depot-specific stress that exacerbates metabolic dysfunction. The dual regulation of hepcidin, systemic via hepatic signaling and local via its expression in the adipose layer, further complicates regional iron trafficking and its metabolic consequences.

Visceral adipose tissue (VAT) is particularly susceptible to iron loading and associated inflammatory responses compared with subcutaneous white adipose tissue (iWAT) and brown adipose tissue (BAT). Several modalities, such as histochemical staining, magnetic resonance imaging, and atomic spectroscopy, consistently show higher levels of labile iron, ferritin, and IL-1β in the VAT [5,145]. This regional enrichment corresponds to increased immune cell infiltration and enhanced NLRP3 inflammasome activation, supporting the role of VAT as a hotspot for metaflammation and insulin resistance [34,41]. While BAT naturally contains more mitochondrial iron because of its oxidative requirements, under physiological conditions, it is protected from oxidative stress by robust antioxidant systems, such as uncoupling protein 1 (UCP1). Nevertheless, chronic iron overload impairs the thermogenic function of BAT and stimulates inflammasome-related signaling, suggesting that metabolically protective fat depots are not immune to iron-induced dysfunction [128,208].

Macrophage polarization also determines the pattern and outcome of depot-specific iron processing. In a low-fat environment, iron-buffered macrophage ferritin-expressing high iron (MFe^hi^) macrophages express high levels of CD163 and FPN, enabling efficient removal of extracellular iron and suppression of oxidative damage [41,80]. In contrast, obesity shifts the macrophage pool toward macrophage ferritin-expressing low iron (MFe^lo^) and metabolically activated macrophages (MMe) phenotypes, which are characterized by decreased FPN expression and increased secretion of pro-inflammatory cytokines [32,209]. This phenotypic reprogramming increases iron transfer from macrophages to adipocytes and exacerbates mitochondrial dysfunction and ROS production. These interactions create a feedback loop in which impaired macrophage buffering capacity increases adipocyte iron loading, further fueling redox stress and inflammation.

The accumulation of iron in adipocytes directly impairs endocrine and energy metabolism. Mechanistically, excess iron downregulates adiponectin and leptin expression through forkhead box protein O1 (FOXO1), C/EBP homologous protein (CHOP), and cAMP response element-binding protein (CREB)-dependent transcriptional repression, respectively [32,142]. In parallel, iron promotes ferroptosis, a regulated form of cell death driven by lipid peroxidation and glutathione depletion, which leads to the release of DAMPs that trigger NLRP3 activation [29,31,145]. Mouse models with adipocyte-specific deletion of FPN or macrophage-specific CD163 deficiency exhibit exaggerated iron retention, increased IL-1β expression, and systemic insulin resistance [32,79,80]. In addition, a high-fat diet (HFD) and exposure to lipopolysaccharide (LPS) induce local hepcidin production in adipose tissues, which further drives FPN degradation and intracellular iron storage [5,84].

Experimental evidence confirms these observations. In vitro co-cultures with isotope-labeled iron show that obesity reduces the efficiency of iron buffering by macrophages, while increasing iron uptake by adipocytes [81]. Importantly, both adipocytes and ATMs contribute to local hepcidin expression, which explains the paradoxical coexistence of systemic hypoferremia and adipose iron overload frequently observed in obesity and T2D [85,87,210]. This local iron retention disrupts mitochondrial respiration not only in adipose tissue but also in metabolically active organs, such as skeletal muscle and pancreatic β-cells, which impair glucose oxidation and increase insulin resistance [128,145,211]. Thus, regional dysregulation of iron balance is a central feature of obesity-related metabolic disorders. To elucidate the complex interactions among iron trafficking, immune cell behavior, and endocrine function, Table 4 presents a comparative summary of the major mechanistic axes implicated in obesity-related metaflammation. These include adipocyte iron overload, ATM–adipocyte iron exchange, and inflammation-driven hepcidin signaling, all of which are linked to discrete molecular mediators and metabolic consequences.

## 7. Gaps in Knowledge and Future Direction

Although considerable progress has been made in mapping the interplay between iron metabolism and lipid-driven inflammation via the NLRP3 inflammasome, the crucial mechanistic, spatial, and temporal questions remain unresolved. Current evidence, largely based on correlations, suggests that regional iron overload in adipose tissue contributes to inflammasome activation and metabolic dysfunction. However, few studies have directly manipulated iron flux to define causality or clarify whether iron promotes the priming or activation phases of NLRP3 signaling. This limitation hampers translational potential and underscores the need for more refined models and targeted biomarkers.

A major gap lies in the lack of depot- and cell-specific mechanistic studies. Most studies rely on bulk tissue analysis, which obscures the heterogeneity of fat deposits, such as VAT, subcutaneous adipose tissue (SAT), and BAT [32,42]. VAT appears to be particularly susceptible to iron-induced inflammasome activity, but there are few comparative studies on fat depots. Although macrophages are important mediators, other cell types including adipocytes, endothelial cells, and fibroblasts also contribute to iron processing and inflammasome regulation. The lack of conditional knockouts targeting key genes such as Nlrp3, Casp1, Hamp1, or Slc40a1 in lipid-relevant cell types further limits the mechanistic clarity. Technological advances such as spatial transcriptomics (e.g., CODEX, MIBI-TOF, Slide-seq), single-cell iron mapping, and live-cell imaging can elucidate these spatial and temporal dynamics. Genetic models, such as Cd163–/– or Nlrp3–/– mice, combined with interventions, such as deferoxamine or deferasirox, in the presence of inflammasome triggers, can clarify the sequence of immune activation and metabolic remodeling. Functional imaging platforms (e.g., iron-sensitive MRI and radiotracers) can track iron flow in vivo and bridge the gap between experimental systems and clinical applications.

However, its clinical implementation is challenging. Ferritin is widely used, but not very specifically, because of its role as a reactant in the acute phase. Composite indices, such as the hepcidin–ferritin ratio or sTfR/log (ferritin), are promising, but need to be validated. In addition, the interaction between iron and inflammation in non-adipose tissues such as skeletal muscle and pancreatic β-cells remains poorly understood, although these tissues are critical for glucose regulation and are susceptible to iron-induced stress.

To address these gaps, future research should prioritize systems relevant to humans, including organoid platforms and adipose tissue biopsies from individuals with obesity or T2D. Integrative multi-omics approaches, along with depot-specific therapeutic trials, may identify actionable targets within the ATM–adipocyte axis and hepcidin–ferroportin signaling network. Novel therapies including nanoparticle-based chelating agents, IL-1β/NLRP3 inhibitors, and depot-specific hepcidin modulators offer promising avenues for precision medicine. Unlocking the translational potential of the iron–inflammasome axis requires interdisciplinary efforts that combine molecular biology, systems modeling, and clinical investigations. Addressing these knowledge gaps is critical for the development of targeted diagnostics and interventions to mitigate inflammation-induced insulin resistance and metabolic disease progression.

## 8. Conclusions

The interplay between iron metabolism and NLRP3 inflammasome signaling represents a critical but poorly understood axis in the pathophysiology of obesity and type 2 diabetes (T2D). There is compelling evidence that impaired iron distribution, particularly the intracellular accumulation of labile iron in adipose tissue, triggers a cascade of sterile inflammatory responses. These responses are mediated by reactive oxygen species (ROS), lipid peroxidation, and ferroptosis, culminating in the activation of IL-1β and the broader inflammasome complex. These processes synergistically impair the endocrine function of adipocytes, alter the immune cell composition, and lead to persistent insulin resistance. Among fat depots, visceral adipose tissue (VAT) exhibits increased sensitivity to iron-induced inflammatory stress owing to its rich immune environment and vascular connection to systemic circulation, which exacerbates its metabolic effects.

Mechanistically, iron-induced oxidative stress promotes the formation of DAMPs and oxidized mitochondrial DNA, both of which act as potent activators of the NLRP3 inflammasome. This activity is amplified by IL-6–induced hepcidin expression, which suppresses ferroportin (FPN) and facilitates further intracellular iron deposition, creating a vicious cycle of metabolic inflammation. These processes are not uniformly distributed across fat depots or cell types, emphasizing the need for spatially refined and temporally resolved mechanistic studies. The interaction between adipocytes and adipose tissue macrophages (ATMs), which is regulated by iron deficiency, modulates depot-specific outcomes and underscores the role of the microenvironment in shaping disease progression.

Although therapeutic strategies targeting iron overload and inflammasome activity, such as deferoxamine, IL-1 blockers, and NLRP3 inhibitors, have shown efficacy in preclinical models, their translation into clinical practice remains limited by issues of specificity, systemic side effects, and insufficient biomarker levels. The development of localized or cell-type selective interventions is promising, but it requires validated diagnostic tools to stratify patients based on iron-processing phenotypes and inflammatory profiles. Advances in single-cell omics, spatial transcriptomics, and noninvasive iron imaging have provided powerful platforms to elucidate these mechanisms in human metabolic tissues, especially when integrated with metabolic flux assays and inflammasome function measurements.

Translational advances also depend on the use of robust biomarker panels, such as the hepcidin-to-ferritin ratio, soluble transferrin receptor levels, and BRINDA project (biomarkers reflecting inflammation and nutritional determinants of anemia)-corrected iron estimates, in metabolic disease studies. These indices, in combination with longitudinal clinical data and intervention outcomes, can serve as a basis for the development of precision strategies targeting iron-related metaflammation. In addition, new models, such as adipose organoids, co-culture systems, and in vivo reporter mice, provide experimental platforms to study depot-specific iron flux and immune–metabolic crosstalk.

Taken together, the iron–inflammasome axis represents a high-value target for mechanism discovery and clinical innovation in metabolic diseases. By deciphering its molecular circuitry and anatomical heterogeneity, future research may lead to stratified therapies that address the dual burden of iron dysregulation and inflammation in obesity and T2D. This paradigm shift from associative observations to mechanistically anchored tissue-specific interventions may pave the way for novel diagnostic and therapeutic strategies with far-reaching implications across the spectrum of immunometabolic disorders.

## Figures and Tables

**Table 1:** Mechanistic pathways linking iron metabolism and inflammation in obesity and T2D.

Aspect/Mechanism	Key Findings and Molecular Pathways	References
Adipocyte iron overload	Elevated intracellular iron in adipocytes suppresses insulin-sensitizing adiponectin (via FOXO1) and leptin (via CREB), promoting insulin resistance and metabolic dysregulation. Iron homeostasis in adipocytes is governed by the hepcidin–ferroportin axis, where both systemic (hepatic, inflammation-induced) and local (adipocyte/macrophage-derived) hepcidin mediate autocrine and paracrine signaling.	[32,36,77,78]
Atm (adipose tissue macrophage) iron handling	Obesity drives macrophage polarization toward pro-inflammatory M1 phenotypes, which exhibit reduced iron content, lower ferroportin expression, and diminished iron recycling. This transition depletes MFe^hi^ (iron-rich, M2-like) macrophages and contributes to iron sequestration, ROS generation, ferroptosis, and amplified adipose tissue inflammation.	[41,79,80,81,82,83]
Inflammation-induced hepcidin expression	IL-6-driven STAT3 activation stimulates hepcidin synthesis in both liver and adipose tissue; this is reinforced by BMP/SMAD signaling. Hepcidin degrades ferroportin, restricting iron export and promoting intracellular iron retention in adipocytes, macrophages, and enterocytes. The result is localized iron overload, impaired insulin signaling, and dysregulated adipokine secretion.	[84,85,86,87]
Iron-driven oxidative stress and ferroptosis	Redox-active Fe^2+^ promotes hydroxyl radical formation via Fenton chemistry, leading to lipid peroxidation and ferroptotic cell death in adipocytes and hepatocytes. In obesity models, ferroptosis inhibition (e.g., by deferoxamine or GPX4 activation) preserves tissue integrity and metabolic function. Iron accumulation in adipose and skeletal muscle correlates with mitochondrial dysfunction and autophagy impairment.	[36,88]
Macrophage–adipocyte iron exchange	Obesity alters ATM iron export/import dynamics, particularly in MMe (metabolically activated) macrophages, enhancing iron transfer to adipocytes. Stable isotope tracing confirms bidirectional iron misdistribution within obese adipose tissue, reinforcing a feedback loop of inflammation and insulin resistance.	[41,81,82,83]
Role of CD163 in hemoglobin clearance and atm phenotype	CD163-deficient models exhibit impaired hemoglobin–haptoglobin scavenging, increased cytokine production, elevated adipocyte iron, and worsened insulin resistance. These findings link iron clearance to ATM polarization and broader metabolic homeostasis.	[79]
Iron-regulated gene networks	Obesity alters expression of iron-handling genes in adipocytes and macrophages, including transferrin, ferritin, ferroportin, DMT1, TfR1, and IRP1/2. Non-canonical regulators of hepcidin—such as leptin and ER stress—may influence local iron retention beyond inflammation alone.	[84,86,89,90]

Abbreviations: ATM, adipose tissue macrophage; BMP, bone morphogenetic protein; CD-163, cluster of differentiation 163; CREB, cAMP response element-binding protein; DMT-1, divalent metal transporter 1; FOXO1, forkhead box protein O1; GPX4, glutathione peroxidase 4; IL-6, interleukin-6; IRP1/2, iron regulatory protein 1 and 2; MFehi, macrophages with high iron content; ROS, reactive oxygen species; SMAD, small mothers against decapentaplegic homolog; STAT3, signal transducer and activator of transcription 3; TfR1, transferrin receptor 1.

**Table 2:** Experimental models and human studies exploring iron–inflammasome dynamics in obesity and T2D.

Model/System	Notable Features and Mechanistic Relevance	References
Mouse genetic models	Conditional knockout models targeting ferroportin in adipocytes, CD163 in macrophages, or systemic/local hepcidin expression provide direct evidence for iron’s causal role in adipocyte–macrophage crosstalk. These models reveal macrophage phenotype shifts (e.g., MFe^hi^ to MMe) and measurable downstream metabolic dysfunction.	[32,41,79,80,81,164]
High-fat diet (HFD) rodents	Chronic HFD feeding induces low-grade metaflammation and iron misdistribution similar to human obesity and T2D. Offers more relevant insights than acute iron-loading or endotoxemia models.	[41,80,84,86,128,165,166]
Iron chelation/loading interventions	Iron chelators (e.g., deferoxamine) lower adipose inflammation, ROS levels, and insulin resistance in obese models. Dietary or parenteral iron loading increases ATM iron burden and modifies inflammatory signaling in VAT.	[36,128,167]
Adipocyte–macrophage co-culture and isotope tracing	In vitro co-culture systems and tracer-based models demonstrate direct, bidirectional iron exchange. Reveal obesity-induced alterations in cell-specific iron flux and retention [32,41,79,80,81,167].	[41,81]
Human adipose tissue and cohort studies	Expression levels of key iron metabolism genes (e.g., ferroportin, hepcidin, ferritin) in adipose tissue correlate with adipokine secretion, insulin resistance, and clinical features of metabolic syndrome. Provide translational linkage to rodent findings.	[32,85,87,89,168]

Abbreviations: ATM, adipose tissue macrophage; CD163, cluster of differentiation 163; HFD, high-fat diet; IL-1β, interleukin-1 beta; KO, knockout; MFehi, macrophages with high iron content; MMe, metabolically activated macrophages; ROS, reactive oxygen species; T2D, type 2 diabetes; VAT, visceral adipose tissue.

**Table 3:** Cell-type and tissue-specific mechanisms linking iron dysregulation to inflammation in obesity and T2D.

Cell Type/Tissue	Mechanistic Insights	References
Adipocytes	Primary sites of iron accumulation and sources of local hepcidin production. Iron suppresses insulin-sensitizing adiponectin (via FOXO1) and leptin (via CREB), impairing endocrine function. Alters metabolic and inflammatory gene expression, disrupts mitochondrial dynamics, and predisposes to ferroptosis.	[32,77,165]
Adipose tissue macrophages (ATMs)	Act as critical iron buffers in lean states (MFe^hi^) but shift toward pro-inflammatory M1/MMe phenotypes under obesity. Exhibit impaired ferroportin expression and reduced iron recycling. ATM iron handling directly governs paracrine iron transfer to adipocytes and the local inflammatory milieu.	[41,79,80,81,82]
Liver/hepatocytes	Central hub for systemic iron regulation through inflammation-induced hepcidin synthesis. Orchestrates whole-body iron homeostasis and storage. Responds to IL-6, BMP/SMAD, and iron-sensing pathways altered in obesity and T2D.	[84,85,86,207]
Skeletal muscle and pancreatic β-cells	Both tissues experience iron-driven oxidative stress and mitochondrial dysfunction in obesity and insulin-resistant states. Typically affected secondarily to adipose dysfunction but contribute to impaired glucose handling.	[88]

Abbreviations: ATM, adipose tissue macrophage; BMP, bone morphogenetic protein; CREB, cAMP response element-binding protein; FOXO1, forkhead box protein O1; IL-6, interleukin-6; M1, classically activated macrophage; MFehi, iron-rich macrophage phenotype; MMe, metabolically activated macrophage; SMAD, small mothers against decapentaplegic homolog; T2D, type 2 diabetes.

**Table 4:** Comparative summary of key mechanistic axes linking iron metabolism to inflammation and insulin resistance.

Axis	Direct Evidence/Experimental Manipulation	Cell/Tissue Specificity	Key Mediators/Pathways	Pathophysiological Outcome	References
Adipocyte iron overload	Genetic models (FPN KO), dietary overload, iron chelation	Adipocytes, adipose macrophages, liver	Ferroportin, hepcidin, FOXO1, CREB	↓ Adiponectin and leptin, ↑ insulin resistance	[32,77,78,212]
Macrophage–adipocyte iron flux	Conditional KO (CD163, FPN), co-culture systems, in vivo iron tracing	MFe^hi^/MMe macrophages, adipocytes	Ferroportin, transferrin, IRE–IRP system	ATM iron loss, adipocyte overload, ↑ inflammation	[41,79,80,82]
Hepcidin induction by IL-6	Obese mouse models, human adipose/liver biopsy data	Liver, adipocytes, stromal vascular fraction (SVF)	IL-6, STAT3, BMP–SMAD signaling	Iron sequestration, intracellular overload, functional systemic deficiency	[84,85,86]
Iron-driven ROS and ferroptosis	Iron overload/supplementation, chelation (DFO), ferroptosis modulators	Adipocytes, hepatocytes, skeletal muscle	Fenton chemistry, GPX4, NRF2, HIF1α	Lipid peroxidation, mitochondrial dysfunction, ↑ insulin resistance	[128,166]
Molecular effects on gene networks	Transcriptomic and proteomic profiling, IRE-regulated gene analysis	Adipocytes, ATMs	Transferrin, ferritin, DMT1, TfR1, hepcidin	Altered insulin sensitivity, disrupted adipokine secretion	[84,86,89,90]

Abbreviations: ATM, adipose tissue macrophage; BMP, bone morphogenetic protein; CD163, cluster of differentiation 163; CREB, cAMP response element-binding protein; DMT1, divalent metal transporter 1; FPN, ferroportin; FOXO1, forkhead box protein O1; GPX4, glutathione peroxidase 4; HIF1α, hypoxia-inducible factor 1-alpha; IL-6, interleukin-6; KO, knockout; NRF2, nuclear factor erythroid 2 related factor 2; SMAD, small mothers against decapentaplegic homolog; STAT3, signal transducer and activator of transcription 3; TfR1, transferrin receptor 1; ↓, decrease; ↑, increase.

## Data Availability

This review does not involve new data collection methods. All referenced materials were obtained from publicly available, peer-reviewed publications. No proprietary or unpublished data were used.

## Abbreviations

The following abbreviations are used in this manuscript:

4-HNE	4-Hydroxynonenal
ACD	Anemia of chronic disease
AI	Anemia of inflammation
AKT/PKB	Protein kinase B
ALAS2	5′-Aminolevulinate synthase 2
AMPK	AMP-activated protein kinase
ASC	Apoptosis-associated speck-like protein containing a CARD
ATMs	Adipose tissue macrophages
ATP	Adenosine triphosphate
BAT	Brown adipose tissue
BMP6	Bone morphogenetic protein 6
BRINDA	Biomarkers reflecting inflammation and nutritional determinants of anemia
C/EBP	CCAAT/enhancer-binding protein
Ca^2+^	Calcium ion
CARD	Caspase recruitment domain
CD11c^+^	Cluster of differentiation 11c positive
CD163	Cluster of differentiation 163
CD4+ T cells	Cluster of differentiation 4 positive T lymphocytes
CHOP	C/EBP homologous protein
Cl^−^	Chloride ion
CLS	Crown-like structures
CODEX	CO-Detection by indEXing
CREB	cAMP response element-binding protein
DAMPs	Damage-associated molecular patterns
Dcytb	Duodenal cytochrome b
DFO	Deferoxamine
DFP	Deferiprone
DFX	Deferasirox
DHA	Docosahexaenoic acid
DIOS	Dysmetabolic iron overload syndrome
DNA	Deoxyribonucleic acid
DMT1	Divalent metal transporter 1
Fe–S	Iron–sulfur cluster
Fe^2+^	Ferrous iron
Fe^3+^	Ferric iron
FBXL5	F-box and leucine-rich repeat protein 5
FOXO1	Forkhead box protein O1
FPN/Ferroportin	Ferroportin (iron exporter), encoded by SLC40A1
GPX4	Glutathione peroxidase 4
GSDMD	Gasdermin D
H_2_O_2_	Hydrogen peroxide
HAMP/HAMP1	Hepcidin antimicrobial peptide (gene)
HCD	High-carbohydrate diet
HERC2	HECT and RLD domain-containing E3 ubiquitin protein ligase 2
HFD	High-fat diet
HFE	High Fe gene (homeostatic iron regulator)
HIF-2α	Hypoxia-inducible factor 2 alpha
HIFs	Hypoxia-inducible factors
HJV	Hemojuvelin
HMGB1	High mobility group box 1
HO-1	Heme oxygenase-1
IAPP	Islet amyloid polypeptide
IL-18	Interleukin-18
IL-1β	Interleukin-1 beta
IL-1R	Interleukin-1 receptor
IL-22	Interleukin-22
IL-6	Interleukin-6
iWAT	Inguinal white adipose tissue (subcutaneous)
IRE/IREs	Iron responsive element (s)
IRP/IRPs	Iron regulatory protein (s)
IRS	Insulin receptor substrate
IRS-1	Insulin receptor substrate 1
JAK–STAT3	Janus kinase–signal transducer and activator of transcription 3
JNK/MAPK	c-Jun N-terminal kinase/mitogen-activated protein kinase
LIP	Labile iron pool
LPS	Lipopolysaccharide
LRR	Leucine-rich repeat
MAPK	Mitogen-activated protein kinase
MAVS	Mitochondrial antiviral signaling protein
MFehi	Macrophage ferritin-expressing high iron phenotype
MFelo	Macrophage ferritin-expressing low iron phenotype
MIBI-TOF	Multiplexed ion beam imaging by time-of-flight
MMe	Metabolically activated macrophage phenotype
MRI	Magnetic resonance imaging
mTOR	Mammalian (mechanistic) target of rapamycin
mtROS	Mitochondria-derived reactive oxygen species
Na^+^	Sodium ion
NCOA4	Nuclear receptor coactivator 4
NEK7	NIMA-related kinase 7
NF-κB	Nuclear factor kappa-light-chain-enhancer of activated B cells
NLRP3	NOD-, LRR-, and pyrin domain-containing protein 3
NRF2	Nuclear factor erythroid 2–related factor 2
NTBI	Non–transferrin-bound iron
PAMPs	Pathogen-associated molecular patterns
PUFAs	Polyunsaturated fatty acids
RAGE	Receptor for advanced glycation end products
ROS	Reactive oxygen species
SAT	Subcutaneous adipose tissue
sTfR	Soluble transferrin receptor
SLC40A1	Solute carrier family 40 member 1 (gene encoding ferroportin)
SOCS	Suppressor of cytokine signaling
T2D	Type 2 diabetes mellitus
Tf/TfR/TfR1	Transferrin/Transferrin receptor/Transferrin receptor 1
THP-1	Human myelomonocytic cell line THP-1
TLR/TLR4	Toll-like receptor/Toll-like receptor 4
TNF-α	Tumor necrosis factor alpha
TRX	Thioredoxin
TXNIP	Thioredoxin-interacting protein
UCP1	Uncoupling protein 1
UTR	Untranslated region
VAT	Visceral adipose tissue
vWAT	Visceral white adipose tissue

## References

[1] GBD 2021 Diabetes Collaborators (2023). Global, regional, and national burden of diabetes from 1990 to 2021, with projections of prevalence to 2050: A systematic analysis for the global burden of disease study 2021. Lancet.

[2] WHO (2025). Obesity and Overweight.

[3] Hotamisligil G.S. (2017). Inflammation, metaflammation and immunometabolic disorders. Nature.

[4] Unamuno X., Gómez-Ambrosi J., Ramírez B., Rodríguez A., Becerril S., Valentí V., Moncada R., Silva C., Salvador J., Frühbeck G. (2019). Nlrp_3_ inflammasome blockade reduces adipose tissue inflammation and extracellular matrix remodeling. Cell. Mol. Immunol..

[5] Hilton C., Sabaratnam R., Drakesmith H., Karpe F. (2023). Iron, glucose and fat metabolism and obesity: An intertwined relationship. Int. J. Obes..

[6] Charles-Messance H., Mitchelson K.A.J., de Marco Castro E., Sheedy F.J., Roche H.M. (2020). Regulating metabolic inflammation by nutritional modulation. J. Allergy Clin. Immunol..

[7] Sharma M., Boytard L., Hadi T., Koelwyn G.J., Simon R., Ouimet M., Seifert L., Spiro W., Yan B., Hutchison S. (2020). Enhanced glycolysis and hif-1α activation in adipose tissue macrophages sustains local and systemic interleukin-1β production in obesity. Sci. Rep..

[8] Fuster J.J., Zuriaga M.A., Zorita V., MacLauchlan S., Polackal M.N., Viana-Huete V., Ferrer-Pérez A., Matesanz N., Herrero-Cervera A., Sano S. (2020). Tet_2_-loss-of-function-driven clonal hematopoiesis exacerbates experimental insulin resistance in aging and obesity. Cell Rep..

[9] Esser N., Legrand-Poels S., Piette J., Scheen A., Paquot N. (2014). Inflammation as a link between obesity, metabolic syndrome and type 2 diabetes. Diabetes Res. Clin. Pract..

[10] Weisberg S.P., McCann D., Desai M., Rosenbaum M., Leibel R.L., Ferrante A.W. (2003). Obesity is associated with macrophage accumulation in adipose tissue. J. Clin. Investig..

[11] Lumeng C.N., Bodzin J.L., Saltiel A.R. (2007). Obesity induces a phenotypic switch in adipose tissue macrophage polarization. J. Clin. Investig..

[12] Hu T.X., Zhang N.N., Ruan Y., Tan Q.Y., Wang J. (2019). Hydrogen sulfide modulates high glucose-induced NLRP3 inflammasome activation in 3T3-L1 adipocytes. Exp. Ther. Med..

[13] McLaughlin T., Liu L.-F., Lamendola C., Shen L., Morton J.M., Rivas H., Winer D.A., Tolentino L.L., Choi O., Zhang H. (2014). T-cell profile in adipose tissue is associated with insulin resistance and systemic inflammation in humans. Arterioscler. Thromb. Vasc. Biol..

[14] Osborn O., Olefsky J.M. (2012). The cellular and signaling networks linking the immune system and metabolism in disease. Nat. Med..

[15] Sun K., Kusminski C.M., Scherer P.E. (2011). Adipose tissue remodeling and obesity. J. Clin. Investig..

[16] Wu K.K.-L., Cheung S.W.-M., Cheng K.K.-Y. (2020). NLRP3 inflammasome activation in adipose tissues and its implications on metabolic diseases. Int. J. Mol. Sci..

[17] Stienstra R., van Diepen J.A., Tack C., Zaki M., van de Veerdonk F.L., Deshani P., Neale G., Hooiveld G., Hijmans A., Vroegrijk I. (2011). Inflammasome is a central player in the induction of obesity and insulin resistance. Proc. Natl. Acad. Sci. USA.

[18] Donath M., Shoelson S. (2011). Type 2 diabetes as an inflammatory disease. Nat. Rev. Immunol..

[19] Tannahill G.M., O’Neill L.A.J. (2011). The emerging role of metabolic regulation in the functioning of toll-like receptors and the nod-like receptor NLRP3. FEBS Lett..

[20] Cho S., Ying F., Sweeney G. (2023). Sterile inflammation and the NLRP3 inflammasome in cardiometabolic disease. Biomed. J..

[21] Paik S., Kim J.K., Silwal P., Sasakawa C., Jo E.-K. (2021). An update on the regulatory mechanisms of nlrp_3_ inflammasome activation. Cell. Mol. Immunol..

[22] Stienstra R., Joosten L.A., Koenen T., van Tits B., van Diepen J.A., van den Berg S.A., Rensen P.C.N., Voshol P.J., Fantuzzi G., Hijmans A. (2010). The inflammasome-mediated caspase-1 activation controls adipocyte differentiation and insulin sensitivity. Cell Metab..

[23] Vandanmagsar B., Youm Y.-H., Ravussin A., Galgani J.E., Stadler K., Mynatt R.L., Ravussin E., Stephens J.M., Dixit V.D. (2011). The NLRP3 inflammasome instigates obesity-induced inflammation and insulin resistance. Nat. Med..

[24] Harris J., Hartman M.L., Roche C.J., Zeng S.G., O’Shea A., Sharp F.A., Lambe E.M., Creagh E.M., Golenbock D.T., Tschopp J. (2011). Autophagy controls il-1β secretion by targeting pro-il-1β for degradation. J. Biol. Chem..

[25] Andrews N.C. (1999). Disorders of iron metabolism. N. Engl. J. Med..

[26] Ganz T., Nemeth E. (2012). Hepcidin and iron homeostasis. Biochim. Biophys. Acta (BBA)-Mol. Cell Res..

[27] Halliwell B., Gutteridge J.M.C. (2015). Free Radicals in Biology and Medicine.

[28] Toyokuni S. (2016). The origin and future of oxidative stress pathology: From the recognition of carcinogenesis as an iron addiction with ferroptosis-resistance to non-thermal plasma therapy. Pathol. Int..

[29] Ma E.B., Javaid H.M.A., Jung D.-H., Park J.-H., Huh J.Y. (2022). Gasdermin d deficiency does not protect mice from high-fat diet-induced glucose intolerance and adipose tissue inflammation. Mediat. Inflamm..

[30] Dixon S.J., Lemberg K.M., Lamprecht M.R., Skouta R., Zaitsev E.M., Gleason C.E., Patel D.N., Bauer A.J., Cantley A.M., Yang W.S. (2012). Ferroptosis: An iron-dependent form of nonapoptotic cell death. Cell.

[31] Fang X., Wang H., Han D., Xie E., Yang X., Wei J., Gu S., Gao F., Zhu N., Yin X. (2019). Ferroptosis as a target for protection against cardiomyopathy. Proc. Natl. Acad. Sci. USA.

[32] Gabrielsen J.S., Gao Y., Simcox J.A., Huang J., Thorup D., Jones D., Cooksey R.C., Gabrielsen D., Adams T.D., Hunt S.C. (2012). Adipocyte iron regulates adiponectin and insulin sensitivity. J. Clin. Investig..

[33] Weiss G., Ganz T., Goodnough L.T. (2019). Anemia of inflammation. Blood.

[34] Simcox J.A., McClain D.A. (2013). Iron and diabetes risk. Cell Metab..

[35] Moreno-Navarrete J., Fernández-Real J. (2023). Iron: The silent culprit in your adipose tissue. Obes. Rev..

[36] Yan H.F., Liu Z.Y., Guan Z.A., Guo C. (2018). Deferoxamine ameliorates adipocyte dysfunction by modulating iron metabolism in ob/ob mice. Endocr. Connect..

[37] Barra N.G., Henriksbo B.D., Anhê F.F., Schertzer J.D. (2020). The nlrp_3_ inflammasome regulates adipose tissue metabolism. Biochem. J..

[38] Rongbin Z., Tardivel A., Thorens B., Choi I., Tschopp J. (2010). Thioredoxin-interacting protein links oxidative stress to inflammasome activation. Nat. Immunol..

[39] Ganz T., Nemeth E. (2015). Iron homeostasis in host defence and inflammation. Nat. Rev. Immunol..

[40] Fernández-Real J., López-Bermejo A., Ricart W. (2002). Cross-talk between iron metabolism and diabetes. Diabetes.

[41] Ameka M.K., Beavers W.N., Shaver C.M., Ware L.B., Kerchberger V.E., Schoenfelt K.Q., Sun L., Koyama T., Skaar E.P., Becker L. (2022). An iron refractory phenotype in obese adipose tissue macrophages leads to adipocyte iron overload. Int. J. Mol. Sci..

[42] Oliveras-Canellas N., Latorre J., Santos-Gonzalez E., Lluch A., Ortega F., Mayneris-Perxachs J., Fernandez-Real J.M., Moreno-Navarrete J.M. (2023). Inflammatory response to bacterial lipopolysaccharide drives iron accumulation in human adipocytes. Biomed. Pharmacother..

[43] Knutson M.D. (2017). Iron transport proteins: Gateways of cellular and systemic iron homeostasis. J. Biol. Chem..

[44] Donath M.Y. (2014). Targeting inflammation in the treatment of type 2 diabetes: Time to start. Nat. Rev. Drug Discov..

[45] Rheinheimer J., de Souza B.M., Cardoso N.S., Bauer A.C., Crispim D. (2017). Current role of the NLRP3 inflammasome on obesity and insulin resistance: A systematic review. Metab. Clin. Exp..

[46] Gong L.L., Zhou H.J., Zhao Q.M., Xu N., Huang F.C., Su L.Y., Li W.L. (2025). Molecular mechanism of NLRP3 inflammasome in inflammatory diseases and tumors. Immun. Inflamm. Dis..

[47] Vandanmagsar B., Youm Y., Ravussin A., Galgani J., Stadler K., Mynatt R., Ravussin E., Stephens J., Dixit V. (2011). NLRP3 inflammasome regulates obesity induced systemic inflammation and insulin signaling (117.4). J. Immunol..

[48] Youm Y., Ayinuer A., Vandanmagsar B., Burk D., Ravussin A., Dixit V. (2011). Elimination of the NLRP3-ASC inflammasome protects against chronic obesity-induced pancreatic damage. Endocrinology.

[49] Davis B.K., Wen H., Ting J.P. (2011). The inflammasome nlrs in immunity, inflammation, and associated diseases. Annu. Rev. Immunol..

[50] Liu T., Zhang L., Joo D., Sun S.-C. (2017). Nf-κb signaling in inflammation. Signal Transduct. Target. Ther..

[51] Duewell P., Kono H., Rayner K.J., Sirois C.M., Vladimer G., Bauernfeind F.G., Abela G.S., Franchi L., Nunez G., Schnurr M. (2010). Nlrp_3_ inflammasomes are required for atherogenesis and activated by cholesterol crystals. Nature.

[52] Bauernfeind F., Bartok E., Rieger A., Franchi L., Núñez G., Hornung V. (2011). Cutting edge: Reactive oxygen species inhibitors block priming, but not activation, of the NLRP3 inflammasome. J. Immunol..

[53] Tschopp J., Schroder K. (2010). NLRP3 inflammasome activation: The convergence of multiple signalling pathways on ROS production?. Nat. Rev. Immunol..

[54] Hornung V., Latz E. (2010). Critical functions of priming and lysosomal damage for NLRP3 activation. Eur. J. Immunol..

[55] Pétrilli V., Papin S., Dostert C., Mayor A., Martinon F., Tschopp J. (2007). Activation of the NALP3 inflammasome is triggered by low intracellular potassium concentration. Cell Death Differ..

[56] Zhou R., Yazdi A.S., Menu P., Tschopp J. (2011). A role for mitochondria in NLRP3 inflammasome activation. Nature.

[57] Wani K., Hind A., Alghamdi A., Sabico S., Al-Daghri N. (2021). Role of NLRP3 inflammasome activation in obesity-mediated metabolic disorders. Int. J. Environ. Res. Public Health.

[58] Shi J., Zhao Y., Wang K., Shi X., Yue W., Huang H., Zhuang Y., Cai T., Wang F., Shao F. (2015). Cleavage of gsdmd by inflammatory caspases determines pyroptotic cell death. Nature.

[59] Nakahira K., Haspel J., Rathinam V., Lee S.-J., Dolinay T., Lam H.C., Englert J.A., Rabinovitch M., Cernadas M., Kim H.P. (2010). Autophagy proteins regulate innate immune response by inhibiting nalp3 inflammasome-mediated mitochondrial DNA release. Nat. Immunol..

[60] Franchi L., Eigenbrod T., Muñoz-Planillo R., Núñez G. (2009). The inflammasome: A caspase-1-activation platform that regulates immune responses and disease pathogenesis. Nat. Immunol..

[61] Martinon F., Mayor A., Tschopp J. (2009). The inflammasomes: Guardians of the body. Annu. Rev. Immunol..

[62] Mariathasan S., Weiss D.S., Newton K., McBride J., O’Rourke K., Roose-Girma M., Lee W.P., Weinrauch Y., Monack D.M., Dixit V.M. (2006). Cryopyrin activates the inflammasome in response to toxins and atp. Nature.

[63] Meier D.T., de Paula Souza J., Donath M.Y. (2025). Targeting the NLRP3 inflammasome-il-1beta pathway in type 2 diabetes and obesity. Diabetologia.

[64] Nițulescu I.M., Ciulei G., Cozma A., Procopciuc L.M., Orășan O.H. (2023). From innate immunity to metabolic disorder: A review of the NLRP3 inflammasome in diabetes mellitus. J. Clin. Med..

[65] Broz P., Dixit V.M. (2016). Inflammasomes: Mechanism of assembly, regulation and signalling. Nat. Rev. Immunol..

[66] Yang Q., Liu R., Yu Q., Bi Y., Liu G. (2019). Metabolic regulation of inflammasomes in inflammation. Immunology.

[67] Dalmas E., Venteclef N., Caer C., Poitou C., Cremer I., Aron-Wisnewsky J., Lacroix-Desmazes S., Bayry J., Kaveri S.V., Clément K. (2014). T cell–derived IL-22 amplifies IL-1β–driven inflammation in human adipose tissue: Relevance to obesity and type 2 diabetes. Diabetes.

[68] Zhan X., Li Q., Xu G., Xiao X., Bai Z. (2023). The mechanism of NLRP3 inflammasome activation and its pharmacological inhibitors. Front. Immunol..

[69] Blevins H.M., Xu Y., Biby S., Zhang S. (2022). The NLRP3 inflammasome pathway: A review of mechanisms and inhibitors for the treatment of inflammatory diseases. Front. Aging Neurosci..

[70] Shinnosuke M., Naoe K., Chikara O., Haruka T., Kurata M., Toshihiro Y., Osawa H., Ayaka N., Zako T., Masumoto J. (2018). Iapp/amylin deposition, which is correlated with expressions of asc and il-1β in β-cells of langerhans’ islets, directly initiates NLRP3 inflammasome activation. Int. J. Immunopathol. Pharmacol..

[71] Zhu P., Zhang J.-J., Cen Y., Yang Y., Wang F., Gu K.-P., Yang H.-T., Wang Y.-Z., Zou Z.-Q. (2022). High endogenously synthesized n-3 polyunsaturated fatty acids in fat-1 mice attenuate high-fat diet-induced insulin resistance by inhibiting nlrp_3_ inflammasome activation via akt/gsk-3beta/txnip pathway. Molecules.

[72] Masters S., Latz E., O’Neill L. (2011). The inflammasome in atherosclerosis and type 2 diabetes. Sci. Transl. Med..

[73] Boutens L., Stienstra R. (2016). Adipose tissue macrophages: Going off track during obesity. Diabetologia.

[74] Karamitsos K., Oikonomou E., Theofilis P., Ikonomidis I., Kassi E., Lambadiari V., Gialafos E., Tsatsaragkou A., Mystakidi V.C., Zisimos K. (2025). The role of NLRP3 inflammasome in type 2 diabetes mellitus and its macrovascular complications. J. Clin. Med..

[75] Meyers A.K., Zhu X. (2020). The NLRP3 inflammasome: Metabolic regulation and contribution to inflammaging. Cells.

[76] Tang Y., Wang D., Zhang H., Zhang Y., Wang J., Qi R., Yang J., Shen H., Xu Y., Li M. (2021). Rapid responses of adipocytes to iron overload increase serum TG level by decreasing adiponectin. J. Cell. Physiol..

[77] Gao Y., Li Z., Gabrielsen J.S., Simcox J.A., Lee S.-H., Jones D., Cooksey B., Stoddard G., Cefalu W.T., McClain D.A. (2015). Adipocyte iron regulates leptin and food intake. J. Clin. Investig..

[78] Zhang Z., Funcke J.-B., Zi Z., Zhao S., Straub L.G., Zhu Y., Zhu Q., Crewe C., An Y.A., Chen S. (2021). Adipocyte iron levels impinge on a fat-gut crosstalk to regulate intestinal lipid absorption and mediate protection from obesity. Cell Metab..

[79] Schleh M.W., Ameka M.K., Rodriguez A.S., Hasty A.H. (2024). Deficiency of the hemoglobin-haptoglobin receptor, CD163, worsens insulin sensitivity in obese male mice. Diabetes.

[80] Orr J.S., Kennedy A., Anderson-Baucum E.K., Webb C.D., Fordahl S.C., Erikson K.M., Zhang Y., Etzerodt A., Moestrup S.K., Hasty A.H. (2014). Obesity alters adipose tissue macrophage iron content and tissue iron distribution. Diabetes.

[81] Hubler M.J., Erikson K.M., Kennedy A., Hasty A.H. (2018). MFe^hi^ adipose tissue macrophages compensate for tissue iron perturbations in mice. Am. J. Physiol. Cell Physiol..

[82] Hubler M., Hasty A., Moestrup S., Etzerodt A. (2017). A regulatory role for “MFe^hi”^ macrophages in adipose tissue iron homeostasis. J. Immunol..

[83] Giovanni P. (2023). New insights into adipose tissue metabolic function and dysfunction. Int. J. Mol. Sci..

[84] Gotardo E., Dos Santos A.N., Miyashiro R.A., Gambero S., Rocha T., Ribeiro M.L., Gambero A. (2013). Mice that are fed a high-fat diet display increased hepcidin expression in adipose tissue. J. Nutr. Sci. Vitaminol..

[85] Bekri S., Gual P., Anty R., Luciani N., Dahman M., Ramesh B., Iannelli A., Staccini–Myx A., Casanova D., Amor I.B. (2006). Increased adipose tissue expression of hepcidin in severe obesity is independent from diabetes and nash. Gastroenterology.

[86] Citelli M., Fonte-Faria T., Nascimento-Silva V., Renovato-Martins M., Silva R., Luna A.S., Silva S.V., Barja-Fidalgo C. (2015). Obesity promotes alterations in iron recycling. Nutrients.

[87] Rodríguez-Mortera R., Caccavello R., Ricardo H., Garay-Sevilla M., Gugliucci A. (2021). Higher hepcidin levels in adolescents with obesity are associated with metabolic syndrome dyslipidemia and visceral fat. Antioxidants.

[88] Ji F., Lee H., Kim J.-H. (2024). Regulation of ferroptosis in obesity: Muscle type-specific effects of dietary restriction and exercise. bioRxiv.

[89] Moreno-Navarrete J., Novelle M.G., Catalán V., Ortega F., Moreno M., Gómez-Ambrosi J., Xifra G., Marta S., Guerra E., Ricart W. (2014). Insulin resistance modulates iron-related proteins in adipose tissue. Diabetes Care.

[90] Chung B., Matak P., McKie A.T., Sharp P. (2007). Leptin increases the expression of the iron regulatory hormone hepcidin in HuH7 human hepatoma cells12. J. Nutr..

[91] Odegaard J.I., Chawla A. (2011). Alternative macrophage activation and metabolism. Annu. Rev. Pathol..

[92] Odegaard J.I., Chawla A. (2008). Mechanisms of macrophage activation in obesity-induced insulin resistance. Nat. Clin. Pract. Endocrinol. Metab..

[93] Stienstra R., Tack C.J., Kanneganti T.-D., Joosten L.A.B., Netea M.G. (2012). The inflammasome puts obesity in the danger zone. Cell Metab..

[94] Koenen T.B., Stienstra R., van Tits L.J.H., Joosten L.A.B., van Velzen J.F., Hijmans A., Pol J.A., van der Vliet J.A., Netea M.G., Tack C.J. (2011). The inflammasome and caspase-1 activation: A new mechanism underlying increased inflammatory activity in human visceral adipose tissue. Endocrinology.

[95] Arkan M.C., Hevener A.L., Greten F.R., Maeda S., Li Z.-W., Long J.M., Wynshaw-Boris A., Poli G., Olefsky J.M., Karin M. (2005). Ikk-beta links inflammation to obesity-induced insulin resistance. Nat. Med..

[96] Tanti J.-F., Ceppo F., Jager J., Berthou F. (2013). Implication of inflammatory signaling pathways in obesity-induced insulin resistance. Front. Endocrinol..

[97] Gora I.M., Ciechanowska A., Ladyzynski P. (2021). NLRP3 inflammasome at the interface of inflammation, endothelial dysfunction, and type 2 diabetes. Cells.

[98] Chiazza F., Couturier-Maillard A., Benetti E., Mastrocola R., Nigro D., Cutrin J., Serpe L., Aragno M., Fantozzi R., Ryffel B. (2015). Targeting the nlrp_3_ inflammasome to reduce diet-induced metabolic abnormalities in mice. Mol. Med..

[99] Samuel V.T., Shulman G.I. (2016). The pathogenesis of insulin resistance: Integrating signaling pathways and substrate flux. J. Clin. Investig..

[100] Maedler K., Sergeev P., Ris F., Oberholzer J., Joller-Jemelka H., Spinas G., Kaiser N., Halban P., Donath M. (2002). Glucose-induced β cell production of il-1β contributes to glucotoxicity in human pancreatic islets. J. Clin. Investig..

[101] Böni-Schnetzler M., Häuselmann S.P., Dalmas E., Meier D.T., Thienel C., Traub S., Schulze F., Steiger L., Dror E., Martin P. (2018). β cell-specific deletion of the il-1 receptor antagonist impairs β cell proliferation and insulin secretion. Cell Rep..

[102] Feng X., Ren W., Tang Y., Wen R., Duan H., Yan L. (2022). Palmitic acid impairs INS-1 cells and alters the global gene expression profile. Cell. Mol. Biol..

[103] Kharroubi I., Ladriere L., Cardozo A.K., Dogusan Z., Cnop M., Eizirik D.L. (2004). Free fatty acids and cytokines induce pancreatic beta-cell apoptosis by different mechanisms: Role of nuclear factor-kappab and endoplasmic reticulum stress. Endocrinology.

[104] Liu D., Cardozo A.K., Darville M.I., Eizirik D.L. (2002). Double-stranded rna cooperates with interferon-gamma and il-1 beta to induce both chemokine expression and nuclear factor-kappa b-dependent apoptosis in pancreatic beta-cells: Potential mechanisms for viral-induced insulitis and beta-cell death in type 1 diabetes mellitus. Endocrinology.

[105] Donath M.Y., Storling J., Maedler K., Mandrup-Poulsen T. (2003). Inflammatory mediators and islet beta-cell failure: A link between type 1 and type 2 diabetes. J. Mol. Med..

[106] Chen C., Cohrs C.M., Stertmann J., Bozsak R., Speier S. (2017). Human beta cell mass and function in diabetes: Recent advances in knowledge and technologies to understand disease pathogenesis. Mol. Metab..

[107] Grant R., Dixit V. (2013). Mechanisms of disease: Inflammasome activation and the development of type 2 diabetes. Front. Immunol..

[108] Osborn O., Sara B., Sánchez-Alavez M., Salomon D., Gram H., Bártfai T. (2008). Treatment with an interleukin 1 beta antibody improves glycemic control in diet-induced obesity. Cytokine.

[109] Wen H., Gris D., Lei Y., Jha S., Zhang L., Huang M.T.-H., Brickey W.J., Ting J.P.-Y. (2011). Fatty acid–induced NLRP3-ASC inflammasome activation interferes with insulin signaling. Nat. Immunol..

[110] Kawai T., Autieri M.V., Scalia R. (2020). Adipose tissue inflammation and metabolic dysfunction in obesity. Am. J. Physiol. Cell Physiol..

[111] Koenen T.B., Stienstra R., van Tits L.J.H., de Graaf J., Stalenhoef A.F.H., Joosten L.A.B., Tack C.J., Netea M.G. (2011). Hyperglycemia activates caspase-1 and txnip-mediated il-1beta transcription in human adipose tissue. Diabetes.

[112] Patsouris D., Li P., Thapar D., Chapman J., Olefsky J.M., Neels J.G. (2008). Ablation of cd11c-positive cells normalizes insulin sensitivity in obese insulin resistant animals. Cell Metab..

[113] Dinarello C.A. (2011). Interleukin-1 in the pathogenesis and treatment of inflammatory diseases. Blood.

[114] Jager J., Grémeaux T., Cormont M., Le Marchand-Brustel Y., Tanti J.-F. (2006). Interleukin-1beta-induced insulin resistance in adipocytes through down-regulation of insulin receptor substrate-1 expression. Endocrinology.

[115] Tilg H., Moschen A.R. (2006). Adipocytokines: Mediators linking adipose tissue, inflammation and immunity. Nat. Rev. Immunol..

[116] Saltiel A.R., Olefsky J.M. (2017). Inflammatory mechanisms linking obesity and metabolic disease. J. Clin. Investig..

[117] Kyohei N., Fujiwara T., Ishii T., Harigae H., Ogasawara K. (2014). Cellular labile iron activates NLRP3 inflammasome. Blood.

[118] Nakamura K., Kawakami T., Yamamoto N., Tomizawa M., Fujiwara T., Ishii T., Harigae H., Ogasawara K. (2015). Activation of the NLRP3 inflammasome by cellular labile iron. Exp. Hematol..

[119] O’Brien-Ladner A.R., Nelson S.R., Murphy W.J., Blumer B.M., Wesselius L.J. (2000). Iron is a regulatory component of human il-1beta production. Support for regional variability in the lung. Am. J. Respir. Cell Mol. Biol..

[120] Ginzburg Y.Z. (2019). Hepcidin-ferroportin axis in health and disease. Vitam. Horm..

[121] Taher Ali T., Musallam Khaled M., Cappellini M.D. (2021). β-thalassemias. N. Engl. J. Med..

[122] Entezari S., Haghi S.M., Norouzkhani N., Sahebnazar B., Vosoughian F., Akbarzadeh D., Islampanah M., Naghsh N., Abbasalizadeh M., Deravi N. (2022). Iron chelators in treatment of iron overload. J. Toxicol..

[123] Rivella S. (2009). Ineffective erythropoiesis and thalassemias. Curr. Opin. Hematol..

[124] Saeed S., Quintin J., Kerstens H.H., Rao N.A., Aghajanirefah A., Matarese F., Cheng S.C., Ratter J., Berentsen K., van der Ent M.A. (2014). Epigenetic programming of monocyte-to-macrophage differentiation and trained innate immunity. Science.

[125] Camaschella C. (2015). Iron-deficiency anemia. N. Engl. J. Med..

[126] Galaris D., Barbouti A., Pantopoulos K. (2019). Iron homeostasis and oxidative stress: An intimate relationship. Biochim. Biophys. Acta (BBA)-Mol. Cell Res..

[127] Xue H., Chen D., Zhong Y., Zhou Z., Fang S., Li M., Guo C. (2016). Deferoxamine ameliorates hepatosteatosis via several mechanisms in ob/ob mice. Ann. N. Y. Acad. Sci..

[128] Tajima S., Ikeda Y., Sawada K., Yamano N., Horinouchi Y., Kihira Y., Ishizawa K., Izawa-Ishizawa Y., Kawazoe K., Tomita S. (2012). Iron reduction by deferoxamine leads to amelioration of adiposity via the regulation of oxidative stress and inflammation in obese and type 2 diabetes kkay mice. Am. J. Physiol. Endocrinol. Metab..

[129] Ni S., Yuan Y., Kuang Y., Li X. (2022). Iron metabolism and immune regulation. Front. Immunol..

[130] Ward R.J., Crichton R.R., Taylor D.L., Corte L.D., Srai S.K., Dexter D.T. (2011). Iron and the immune system. J. Neural Transm..

[131] Legrand-Poels S., Esser N., L’Homme L., Scheen A., Paquot N., Piette J. (2014). Free fatty acids as modulators of the NLRP3 inflammasome in obesity/type 2 diabetes. Biochem. Pharmacol..

[132] Martínez-Micaelo N., González-Abuín N., Pinent M., Ardévol A., Blay M. (2016). Dietary fatty acid composition is sensed by the NLRP3 inflammasome: Omega-3 fatty acid (dha) prevents NLRP3 activation in human macrophages. Food Funct..

[133] Reynolds C., McGillicuddy F., Karen A.H., Finucane O., Mills K., Roche H. (2012). Dietary saturated fatty acids prime the NLRP3 inflammasome via TLR4 in dendritic cells-implications for diet-induced insulin resistance. Mol. Nutr. Food Res..

[134] Huang Y., Xu W., Zhou R. (2021). NLRP3 inflammasome activation and cell death. Cell Mol. Immunol..

[135] Abderrazak A., Syrovets T., Couchie D., Hadri K.E., Friguet B., Simmet T., Rouis M. (2015). NLRP3 inflammasome: From a danger signal sensor to a regulatory node of oxidative stress and inflammatory diseases. Redox Biol..

[136] Chen X., Yu C., Kang R., Tang D. (2020). Iron metabolism in ferroptosis. Front. Cell Dev. Biol..

[137] Fang X., Ardehali H., Min J., Wang F. (2023). The molecular and metabolic landscape of iron and ferroptosis in cardiovascular disease. Nat. Rev. Cardiol..

[138] Gan B. (2022). Acsl4, pufa, and ferroptosis: New arsenal in anti-tumor immunity. Signal Transduct. Target. Ther..

[139] Yang W.S., Stockwell B.R. (2016). Ferroptosis: Death by lipid peroxidation. Trends Cell Biol..

[140] Koppenol W.H., Hider R.H. (2019). Iron and redox cycling. Do’s and don’ts. Free Radic. Biol. Med..

[141] Kelley N., Jeltema D., Duan Y., He Y. (2019). The NLRP3 inflammasome: An overview of mechanisms of activation and regulation. Int. J. Mol. Sci..

[142] Gao J., Sang M., Zhang X., Zheng T., Pan J., Dai M., Zhou L., Yang S. (2015). Miro1-mediated mitochondrial dysfunction under high nutrient stress is linked to nod-like receptor 3 (NLRP3)-dependent inflammatory responses in rat pancreatic beta cells. Free Radic. Biol. Med..

[143] Stockwell B.R., Friedmann Angeli J.P., Bayir H., Bush A.I., Conrad M., Dixon S.J., Fulda S., Gascón S., Hatzios S.K., Kagan V.E. (2017). Ferroptosis: A regulated cell death nexus linking metabolism, redox biology, and disease. Cell.

[144] Xie Y., Kang R., Klionsky D.J., Tang D. (2023). Gpx4 in cell death, autophagy, and disease. Autophagy.

[145] Zhang S., Sun Z., Jiang X., Lu Z., Ding L., Li C., Tian X., Wang Q. (2022). Ferroptosis increases obesity: Crosstalk between adipocytes and the neuroimmune system. Front. Immunol..

[146] Wen Q., Liu J., Kang R., Zhou B., Tang D. (2019). The release and activity of hmgb1 in ferroptosis. Biochem. Biophys. Res. Commun..

[147] Tang D., Chen X., Kang R., Kroemer G. (2021). Ferroptosis: Molecular mechanisms and health implications. Cell Res..

[148] Chen X., Kang R., Kroemer G., Tang D. (2021). Ferroptosis in infection, inflammation, and immunity. J. Exp. Med..

[149] Ru Q., Li Y., Chen L., Wu Y., Min J., Wang F. (2024). Iron homeostasis and ferroptosis in human diseases: Mechanisms and therapeutic prospects. Signal Transduct. Target. Ther..

[150] Conrad M., Pratt D.A. (2019). The chemical basis of ferroptosis. Nat. Chem. Biol..

[151] Stockwell B.R., Jiang X. (2020). The chemistry and biology of ferroptosis. Cell Chem. Biol..

[152] Katsarou A., Moustakas I.I., Pyrina I., Lembessis P., Koutsilieris M., Chatzigeorgiou A. (2020). Metabolic inflammation as an instigator of fibrosis during non-alcoholic fatty liver disease. World J. Gastroenterol..

[153] Muckenthaler M.U., Rivella S., Hentze M.W., Galy B. (2017). A red carpet for iron metabolism. Cell.

[154] Mancias J.D., Wang X., Gygi S.P., Harper J.W., Kimmelman A.C. (2014). Quantitative proteomics identifies ncoa4 as the cargo receptor mediating ferritinophagy. Nature.

[155] Arosio P., Elia L., Poli M. (2017). Ferritin, cellular iron storage and regulation. IUBMB Life.

[156] Arosio P., Levi S. (2010). Cytosolic and mitochondrial ferritins in the regulation of cellular iron homeostasis and oxidative damage. Biochim. Biophys. Acta (BBA)-Gen. Subj..

[157] Camaschella C., Nai A., Silvestri L. (2020). Iron metabolism and iron disorders revisited in the hepcidin era. Haematologica.

[158] Galy B., Conrad M., Muckenthaler M. (2024). Mechanisms controlling cellular and systemic iron homeostasis. Nat. Rev. Mol. Cell Biol..

[159] Santana-Codina N., Mancias J.D. (2018). The role of ncoa4-mediated ferritinophagy in health and disease. Pharmaceuticals.

[160] Katsarou A., Pantopoulos K. (2020). Basics and principles of cellular and systemic iron homeostasis. Mol. Asp. Med..

[161] Ginzburg Y., An X., Rivella S., Goldfarb A. (2023). Normal and dysregulated crosstalk between iron metabolism and erythropoiesis. eLife.

[162] Maio N., Zhang D.-L., Ghosh M.C., Jain A., SantaMaria A.M., Rouault T.A. (2021). Mechanisms of cellular iron sensing, regulation of erythropoiesis and mitochondrial iron utilization. Semin. Hematol..

[163] Wu K., Zhao W., Hou Z., Zhang W., Qin L., Qiu J., Wang D., Zhuang L., Xue X., Sun D. (2025). Ferritinophagy: Multifaceted roles and potential therapeutic strategies in liver diseases. Front. Cell Dev. Biol..

[164] James J.V., Varghese J., John N.M., Deschemin J.-C., Vaulont S., McKie A.T., Jacob M. (2021). Insulin resistance and adipose tissue inflammation induced by a high-fat diet are attenuated in the absence of hepcidin. J. Nutr. Biochem..

[165] Ma X., Pham V.T., Mori H., MacDougald O.A., Shah Y.M., Bodary P.F. (2017). Iron elevation and adipose tissue remodeling in the epididymal depot of a mouse model of polygenic obesity. PLoS ONE.

[166] Ji F., Lee H., Rheem H., Liu J., Kim J.H. (2025). Differential ferroptosis regulation in red and white gastrocnemius under obesity and its attenuation by exercise and dietary restriction. Sci. Rep..

[167] Gotardo É.M.F., Caria C.R.E.P., de Oliveira C.C., Rocha T., Ribeiro M.L., Gambero A. (2016). Effects of iron supplementation in mice with hypoferremia induced by obesity. Exp. Biol. Med..

[168] Wlazlo N., van Greevenbroek M.M., Ferreira I., Jansen E.H., Feskens E.J., van der Kallen C.J., Schalkwijk C.G., Bravenboer B., Stehouwer C.D. (2013). Iron metabolism is associated with adipocyte insulin resistance and plasma adiponectin. Diabetes Care.

[169] Kruszewski M. (2003). Labile iron pool: The main determinant of cellular response to oxidative stress. Mutat. Res..

[170] Koorts A.M., Viljoen M. (2007). Ferritin and ferritin isoforms i: Structure–function relationships, synthesis, degradation and secretion. Arch. Physiol. Biochem..

[171] Cairo G., Recalcati S., Mantovani A., Locati M. (2011). Iron trafficking and metabolism in macrophages: Contribution to the polarized phenotype. Trends Immunol..

[172] Muckenthaler M., Galy B., Hentze M. (2008). Systemic iron homeostasis and the iron-responsive element/iron-regulatory protein (ire/irp) regulatory network. Annu. Rev. Nutr..

[173] Rouault T.A., Klausner R.D. (1996). Post-transcriptional regulation of genes of iron metabolism in mammalian cells. JBIC J. Biol. Inorg. Chem..

[174] Silva B., Faustino P. (2015). An overview of molecular basis of iron metabolism regulation and the associated pathologies. Biochim. Et Biophys. Acta (BBA)-Mol. Basis Dis..

[175] Anderson G.J., Frazer D.M. (2017). Current understanding of iron homeostasis. Am. J. Clin. Nutr..

[176] Hentze M.W., Muckenthaler M.U., Galy B., Camaschella C. (2010). Two to tango: Regulation of mammalian iron metabolism. Cell.

[177] Mayka S., Galy B., Bjoern S., Jonathon B., Tomi B.-I., Beneš V., Selbach M., Muckenthaler M., Hentze M. (2011). Iron regulatory protein-1 and -2: Transcriptome-wide definition of binding mrnas and shaping of the cellular proteome by iron regulatory proteins. Blood.

[178] Eisenstein R.S., Blemings K.P. (1998). Iron regulatory proteins, iron responsive elements and iron homeostasis12. J. Nutr..

[179] Christine W., Insiya F., Cowan J. (2018). Iron-sulfur cluster biosynthesis and trafficking-impact on human disease conditions. Met. Integr. Biometal Sci..

[180] Anderson C.P., Shen M., Eisenstein R.S., Leibold E.A. (2012). Mammalian iron metabolism and its control by iron regulatory proteins. Biochim. Biophys. Acta (BBA)-Mol. Cell Res..

[181] Wang H., Shi H., Rajan M., Canarie E.R., Hong S., Simoneschi D., Pagano M., Bush M.F., Stoll S., Leibold E.A. (2020). Fbxl5 regulates irp2 stability in iron homeostasis via an oxygen-responsive [2fe2s] cluster. Mol. Cell.

[182] Nemeth E., Ganz T. (2021). Hepcidin-ferroportin interaction controls systemic iron homeostasis. Int. J. Mol. Sci..

[183] Shah Y.M., Matsubara T., Ito S., Yim S.H., Gonzalez F.J. (2009). Intestinal hypoxia-inducible transcription factors are essential for iron absorption following iron deficiency. Cell Metab..

[184] Andrew J.S., Nupur K.D., Ramakrishnan S., Chesta J., Mladen T.J., Jun W., Nemeth E., Lakhal-Littleton S., Colacino J., Shah Y. (2018). Hepatic hepcidin/intestinal hif-2&agr; axis maintains iron absorption during iron deficiency and overload. J. Clin. Investig..

[185] Wilkinson N., Pantopoulos K. (2013). Irp1 regulates erythropoiesis and systemic iron homeostasis by controlling hif2α mrna translation. Blood.

[186] Gulec S., Anderson G.J., Collins J.F. (2014). Mechanistic and regulatory aspects of intestinal iron absorption. Am. J. Physiol. Gastrointest. Liver Physiol..

[187] Ganz T. (2019). Erythropoietic regulators of iron metabolism. Free Radic. Biol. Med..

[188] Park C.H., Valore E.V., Waring A.J., Ganz T. (2001). Hepcidin, a urinary antimicrobial peptide synthesized in the liver. J. Biol. Chem..

[189] Krause A., Neitz S., Mägert H.J., Schulz A., Forssmann W.G., Schulz-Knappe P., Adermann K. (2000). Leap-1, a novel highly disulfide-bonded human peptide, exhibits antimicrobial activity. FEBS Lett..

[190] Nemeth E., Tuttle M.S., Powelson J., Vaughn M.B., Donovan A., Ward D.M., Ganz T., Kaplan J. (2004). Hepcidin regulates cellular iron efflux by binding to ferroportin and inducing its internalization. Science.

[191] Ganz T. (2011). Hepcidin and iron regulation, 10 years later. Blood.

[192] Kautz L., Meynard D., Monnier A., Darnaud V., Bouvet R., Wang R.H., Deng C., Vaulont S., Mosser J., Coppin H. (2008). Iron regulates phosphorylation of smad1/5/8 and gene expression of bmp6, smad7, id1, and atoh8 in the mouse liver. Blood.

[193] Nemeth E., Ganz T. (2009). The role of hepcidin in iron metabolism. Acta Haematol..

[194] Anderson G.J., Vulpe C.D. (2009). Mammalian iron transport. Cell. Mol. Life Sci..

[195] Cani P.D., Bibiloni R., Knauf C., Waget A., Neyrinck A.M., Delzenne N.M., Burcelin R. (2008). Changes in gut microbiota control metabolic endotoxemia-induced inflammation in high-fat diet-induced obesity and diabetes in mice. Diabetes.

[196] Miele L., Valenza V., La Torre G., Montalto M., Cammarota G., Ricci R., Masciana R., Forgione A., Gabrieli M.L., Perotti G. (2009). Increased intestinal permeability and tight junction alterations in nonalcoholic fatty liver disease. Hepatology.

[197] Malesza I.J., Bartkowiak-Wieczorek J., Winkler-Galicki J., Nowicka A., Dzięciołowska D., Błaszczyk M., Gajniak P., Słowińska K., Niepolski L., Walkowiak J. (2022). The dark side of iron: The relationship between iron, inflammation and gut microbiota in selected diseases associated with iron deficiency anaemia-a narrative review. Nutrients.

[198] Yilmaz B., Li H. (2018). Gut microbiota and iron: The crucial actors in health and disease. Pharmaceuticals.

[199] Frawley E.R., Fang F.C. (2014). The ins and outs of bacterial iron metabolism. Mol. Microbiol..

[200] Drakesmith H., Prentice A.M. (2012). Hepcidin and the iron-infection axis. Science.

[201] Soares M.P., Weiss G. (2015). The iron age of host-microbe interactions. EMBO Rep..

[202] Theurl I., Aigner E., Theurl M., Nairz M., Seifert M., Schroll A., Sonnweber T., Eberwein L., Witcher D.R., Murphy A.T. (2009). Regulation of iron homeostasis in anemia of chronic disease and iron deficiency anemia: Diagnostic and therapeutic implications. Blood.

[203] Kanamori Y., Murakami M., Sugiyama M., Hashimoto O., Matsui T., Funaba M. (2017). Interleukin-1β (il-1β) transcriptionally activates hepcidin by inducing ccaat enhancer-binding protein δ (c/ebpδ) expression in hepatocytes. J. Biol. Chem..

[204] Kell D., Pretorius E. (2014). Serum ferritin is an important inflammatory disease marker, as it is mainly a leakage product from damaged cells. Metallomics.

[205] Weiss G., Goodnough L.T. (2005). Anemia of chronic disease. N. Engl. J. Med..

[206] Theurl I., Ludwiczek S., Eller P., Seifert M., Artner E., Brunner P., Weiss G. (2005). Pathways for the regulation of body iron homeostasis in response to experimental iron overload. J. Hepatol..

[207] Nitin P., Yevgeniy A., Chao R., Sacerdoti D., Hibba C., Nichols A., Krithika S., Athar N., Sharma D., Lakhani V. (2017). Heme oxygenase induction suppresses hepatic hepcidin and rescues ferroportin and ferritin expression in obese mice. J. Nutr. Metab..

[208] Guo D.H., Yamamoto M., Hernandez C.M., Khodadadi H., Baban B., Stranahan A.M. (2020). Visceral adipose nlrp_3_ impairs cognition in obesity via il-1r1 on cx3cr1+ cells. J. Clin. Investig..

[209] Jais A., Einwallner E., Sharif O., Gossens K., Lu T.T.H., Soyal S.M., Medgyesi D., Neureiter D., Paier-Pourani J., Dalgaard K. (2014). Heme oxygenase-1 drives metaflammation and insulin resistance in mouse and man. Cell.

[210] Valenti L., Corradini E., Adams L.A., Aigner E., Alqahtani S., Arrese M., Bardou-Jacquet E., Bugianesi E., Fernandez-Real J.M., Girelli D. (2023). Consensus statement on the definition and classification of metabolic hyperferritinaemia. Nat. Rev. Endocrinol..

[211] Heslin A.M., Donnell A.O., Buffini M., Nugent A., Walton J., Flynn A., McNulty B. (2020). Excessive adiposity is associated with an inflammation induced elevation in serum hepcidin, serum ferritin and increased risk of iron overload. Proc. Nutr. Soc..

[212] Ameka M.K., Hasty A.H. (2020). Fat and iron don’t mix. Immunometabolism.

